# Salivary metabolites associated with a 5-year tooth loss identified in a population-based setting

**DOI:** 10.1186/s12916-021-02035-z

**Published:** 2021-07-14

**Authors:** Leonie Andörfer, Birte Holtfreter, Stefan Weiss, Rutger Matthes, Vinay Pitchika, Carsten Oliver Schmidt, Stefanie Samietz, Gabi Kastenmüller, Matthias Nauck, Uwe Völker, Henry Völzke, Laszlo N. Csonka, Karsten Suhre, Maik Pietzner, Thomas Kocher

**Affiliations:** 1 Department of Restorative Dentistry, Periodontology, Endodontology, and Preventive and Pediatric Dentistry, University Medicine Greifswald, Fleischmannstr. 42, 17475 Greifswald, Germany; 2 Interfaculty Institute for Genetics and Functional Genomics, University Medicine Greifswald, Greifswald, Germany; 3 DZHK (German Center for Cardiovascular Research), partner site Greifswald, Greifswald, Germany; 4 Institute for Community Medicine, SHIP/Clinical Epidemiology Research, University Medicine Greifswald, Greifswald, Germany; 5 Department of Prosthetic Dentistry, Gerodontology and Biomaterials, University Medicine Greifswald, Greifswald, Germany; 6 Institute of Bioinformatics and Systems Biology, Helmholtz Zentrum München, Neuherberg, Germany; 7 Institute of Clinical Chemistry and Laboratory Medicine, University Medicine Greifswald, Greifswald, Germany; 8 Department of Biological Sciences, Purdue University, West Lafayette, USA; 9 Weill Cornell Medicine–Qatar, Education City, Qatar Foundation, Doha, Qatar; 10 Computational Medicine, Berlin Institute of Health (BIH), Charité-Universitätsmedizin Berlin, Berlin, Germany

**Keywords:** Metabolomics, Periodontitis, Progression, Tooth loss, 2-Pyrrolidineacetic acid, Butyrylputrescine, Cohort study

## Abstract

**Background:**

Periodontitis is among the most common chronic diseases worldwide, and it is one of the main reasons for tooth loss. Comprehensive profiling of the metabolite content of the saliva can enable the identification of novel pathways associated with periodontitis and highlight non-invasive markers to facilitate time and cost-effective screening efforts for the presence of periodontitis and the prediction of tooth loss.

**Methods:**

We first investigated cross-sectional associations of 13 oral health variables with saliva levels of 562 metabolites, measured by untargeted mass spectrometry among a sub-sample (*n* = 938) of the Study of Health in Pomerania (SHIP-2) using linear regression models adjusting for common confounders. We took forward any candidate metabolite associated with at least two oral variables, to test for an association with a 5-year tooth loss over and above baseline oral health status using negative binomial regression models.

**Results:**

We identified 84 saliva metabolites that were associated with at least one oral variable cross-sectionally, for a subset of which we observed robust replication in an independent study. Out of 34 metabolites associated with more than two oral variables, baseline saliva levels of nine metabolites were positively associated with a 5-year tooth loss. Across all analyses, the metabolites 2-pyrrolidineacetic acid and butyrylputrescine were the most consistent candidate metabolites, likely reflecting oral dysbiosis. Other candidate metabolites likely reflected tissue destruction and cell proliferation.

**Conclusions:**

Untargeted metabolic profiling of saliva replicated metabolic signatures of periodontal status and revealed novel metabolites associated with periodontitis and future tooth loss.

**Supplementary Information:**

The online version contains supplementary material available at 10.1186/s12916-021-02035-z.

## Background

Periodontitis is the sixth-most prevalent health condition, affecting 743 million people worldwide [1], and is associated with severe comorbidities, such as cardiovascular disease, diabetes mellitus, rheumatoid arthritis, and metabolic syndrome [2–6], as well as impairments in the quality of life. Periodontitis is a silent disease without grave symptoms, and the associated socioeconomic burden is substantial [7]. Despite this burden, periodontitis remains understudied and there is a clear call for action by health authorities [8, 9]. Non-invasive techniques such as the molecular characterization of saliva samples can help to identify early disease markers and provide insights into underlying pathological pathways.

Periodontitis is a multifactorial inflammatory disease driven by a dysbiotic microbiome, eliciting host defense mechanisms through interactions of pathogen-associated molecular patterns. The oral cavity can be colonized by over 700 different microbial species [10] and at least 400 species can be found in the subgingival microbiome [11]. Following continuous stimulation of inflammation mediators by subgingival periodontal pathogens, chemotactic recruitment of immune system cells increases. The destruction of periodontal tissues is a consequence of the dysregulated immune response [12, 13].

Up to now, reliable biomarkers for periodontitis do not exist; therefore, developing a robust, consistent, and distinct screening tool to detect periodontitis or to improve the management of periodontitis is of high priority. The saliva is an easily sampled body fluid that has been used to map metabolic events and to survey changes in biological processes in periodontitis [14]. Metabolome profiling by mass spectrometry approaches enables a deep characterization of processes driving the progression of a microbial dysbiosis and possibly adverse host responses [15]. Previous studies already highlighted distinct pathways associated with severe cases of periodontitis [16–20], and we have shown in a large cross-sectional, population-based study that phenylacetate was consistently associated with various periodontitis variables [16]. However, previous studies were limited in their cross-sectional design to disentangle whether the highlighted pathways were cause or consequence of the disease.

In the middle-aged and older persons, periodontitis increases the risk for tooth loss [21, 22]. Persons with periodontitis are three times more likely to lose teeth compared to periodontally healthy persons [23] and, vice versa, persons with future tooth loss display a higher level of periodontal destruction at baseline [21]. In the German adult population, periodontitis is responsible for about 30 to 40% of dental extractions [24]. We chose tooth loss as the primary endpoint for our longitudinal analysis as it presents the most severe outcome for patients and wanted to know if biologically grounded associations of periodontitis with metabolites translate into significant associations between metabolites and tooth loss over and above current clinical measures.

Here, we systematically characterize the molecular fingerprint of current and future oral health status among 938 participants of the population-based Study of Health in Pomerania (SHIP-2) using non-targeted metabolomic analysis covering more than 500 metabolites. We show numerous associations between oral phenotypes and saliva metabolites and replicate previous findings [16–20]. We considerably extend current evidence by identifying a set of metabolites, which were statistically significantly associated with a 5-year tooth loss over and above oral health status at baseline. Among these metabolites, butyrylputrescine and 2-pyrrolidineacetic acid (2-pyrr), putatively of microbial origin, might be promising candidates to be evaluated in future studies.

## Methods

### Study design

The Study of Health in Pomerania (SHIP) is a population-based cohort study in the north-eastern part of Germany [25]. Using a two-stage cluster sampling procedure [26], 7006 Caucasian subjects were randomly drawn, proportional to community population size, and stratified by age and gender. Of 6265 eligible subjects, 4308 participated between 1997 and 2001 (SHIP-0, response 68.8%). Follow-up examinations were conducted after 5 (SHIP-1), 11 (SHIP-2), and 16 years (SHIP-3) [27].

For the present study, we used data from SHIP-2 and SHIP-3. In total, 2333 subjects aged 30 to 93 years participated in SHIP-2. Among these subjects, we selected 1000 participants, who had dental follow-up data in SHIP-3, for metabolic profiling of saliva samples using the Metabolon HD4 platform (Metabolon Inc., Durham, USA). Out of those, we excluded 22 participants whose saliva measurements did not pass the quality control and another 40 participants due to missing values in variables considered as confounders in the statistical analysis. The final data set for statistical analysis included up to 938 individuals (depending on the oral health variable).

### Oral examination

Examinations were conducted in a dental chair with light and without saliva ejector or air jet. Using the half-mouth method (i.e., alternating on the left or right side), periodontal probing depth (PPD) and clinical attachment level (CAL) were assessed on four sites (mesiobuccal, midbuccal, distobuccal, and midpalatinal/midlingual) of all teeth excluding the third molars using a periodontal probe (PCP-11, Hu-Friedy, Chicago, IL, USA). The presence of supragingival plaque and supragingival calculus was recorded on the same four sites at the first incisor, canine, and first molar. If a tooth was missing, the next distally located tooth was considered. The number of teeth was registered excluding wisdom teeth. A dental interview completed the measures.

Coronal cavities were diagnosed visually or using a periodontal probe (PCP-11, Hu-Friedy, Chicago, IL, USA) [28]. Carious defects, fillings, secondary caries, and missing teeth were registered by surface (occlusal, mesial, distal, vestibular, and oral) with the exception of wisdom teeth using the half-mouth method (alternating on the left or right side). The number (DF-S) and the percentage of decayed or filled surfaces (DF-S%) were based on a maximum of 14 permanent teeth (excluding third molars), resulting in 64 surfaces being assessed in total. Information on the presence of removable prostheses in the upper or lower jaw was retrieved from the dental interview.

Five calibrated and licensed dentists performed the dental examinations. Calibration exercises were conducted every 6–12 months. In SHIP-2, intra-class correlations of 0.76–0.88 per examiner and an inter-class correlation of 0.74 for CAL were achieved. For PPD, the intra-class correlation was 0.70–0.78 per examiner and the inter-class correlation was 0.70. In SHIP-3, intra-class correlations of 0.92–0.97 per examiner and an inter-class correlation of 0.98 for CAL were achieved. For PPD, the intra-class correlation was 0.69–0.92 per examiner and the inter-class correlation was 0.91. For caries assessments in SHIP-2, intra-rater kappas were 0.83–1.00, while pairwise inter-rater kappas were 0.72–1.00.

For cross-sectional analyses, the following oral examination variables were derived: mean PPD, cumulative PPD from pockets with PPD ≥4 mm (CumPPD4+), percentage of sites with PPD ≥3 (PPD 3+mm%) or 4 mm (PPD 4+mm%), mean CAL, percentage of sites with CAL ≥3 (CAL 3+mm%) or 4 mm (CAL 4+mm%), number of missing teeth (MT count), percentage of sites with plaque, percentage of sites with calculus, a variable combining information on removable prostheses and MT count (Prosthesis/MT), DF-S, and DF-S%.

Probably due to methodological problems associated with the periodontal probe [29], distributions of periodontal variables, which were based on PPD and CAL measures, were implausibly shifted to the left, indicating on average an improvement of the periodontal status. This was contradicting the fact that about 30% of participants lost at least one tooth. Thus, we renounced from the analysis of the progression of periodontitis variables and restricted longitudinal analyses to a 5-year change in the number of teeth as the outcome.

### Covariates

Sociodemographic and behavioral parameters have been assessed by computer-assisted personal interviews. The smoking status was classified as never, former, and current smoking. Standardized measurements of the body height and weight were performed with calibrated scales, and the body mass index (BMI) was calculated as weight divided by height^2^ (kg/m^2^). Diabetes mellitus was defined as having known diabetes in SHIP-2 or glycated hemoglobin level of (HbA1c) ≥ 6.5% or non-fasting serum glucose ≥11.1 mmol/l. Known diabetes mellitus in SHIP-2 was defined as physician’s diagnosis or antidiabetic medication intake (Anatomic Therapeutic Chemical classification system; code A10) either in SHIP-0, SHIP-1, or SHIP-2. The follow-up time of the examinations was indicated in years.

### Saliva samples

For the collection of saliva samples, a hygienic extraction system (Salivette®; Sarstedt, Nürnbrecht, Germany) was used. The subjects were instructed to chew on a plain cotton roll to stimulate salivation. After exactly 1 min, the rolls containing the absorbed saliva were placed into the Salivette® and centrifuged immediately at 1000 g for 20 min at 4°C. The ejection of food remnants, cell debris, and insoluble materials led to a supernatant, which was stored at −80°C until further analysis.

Metabolic profiling of saliva samples was conducted by Metabolon Inc. (Hallbergmoos, Germany) using the Metabolon Discovery HD4 platform. For each sample, four different ultra-high-performance liquid chromatography-tandem mass spectrometry (UPLC-MS/MS) methods were applied to allow for maximum coverage of the small molecule content. Metabolon uses an internal reference library with more than 3000 pure chemical compounds to identify metabolites based on retention time/index, mass to charge ratio, and chromatographic data. All named compounds fulfill tier 1 or tier 2 (indicated by a star) criteria according to the metabolomics reporting standards [30]. Additional mass spectral entries have been created for structurally unnamed biochemicals, which have been identified by virtue of their recurrent nature (both chromatographic and mass spectral). We accounted for daily variation in instrumental performance by applying a run day-median normalization and transformed normalized intensities using the logarithm to the base 2. To account for different dilutions of saliva samples, we applied probabilistic quotient normalization by estimating a dilution factor for each saliva sample based on 174 metabolites common to all samples [16]. We used the Mahalanobis distance on a reduced metabolic space defined by the first ten principal components of the saliva metabolite data to identify saliva samples differing more than three standard deviations from the multi-dimensional mean.

After processing, 562 saliva metabolites remained for statistical analyses. Out of these, 64 metabolites could not be unambiguously assigned to a chemical identity and are designated to hereafter with an X, combined with a unique number.

### Statistical analysis

**Fig. 1:**
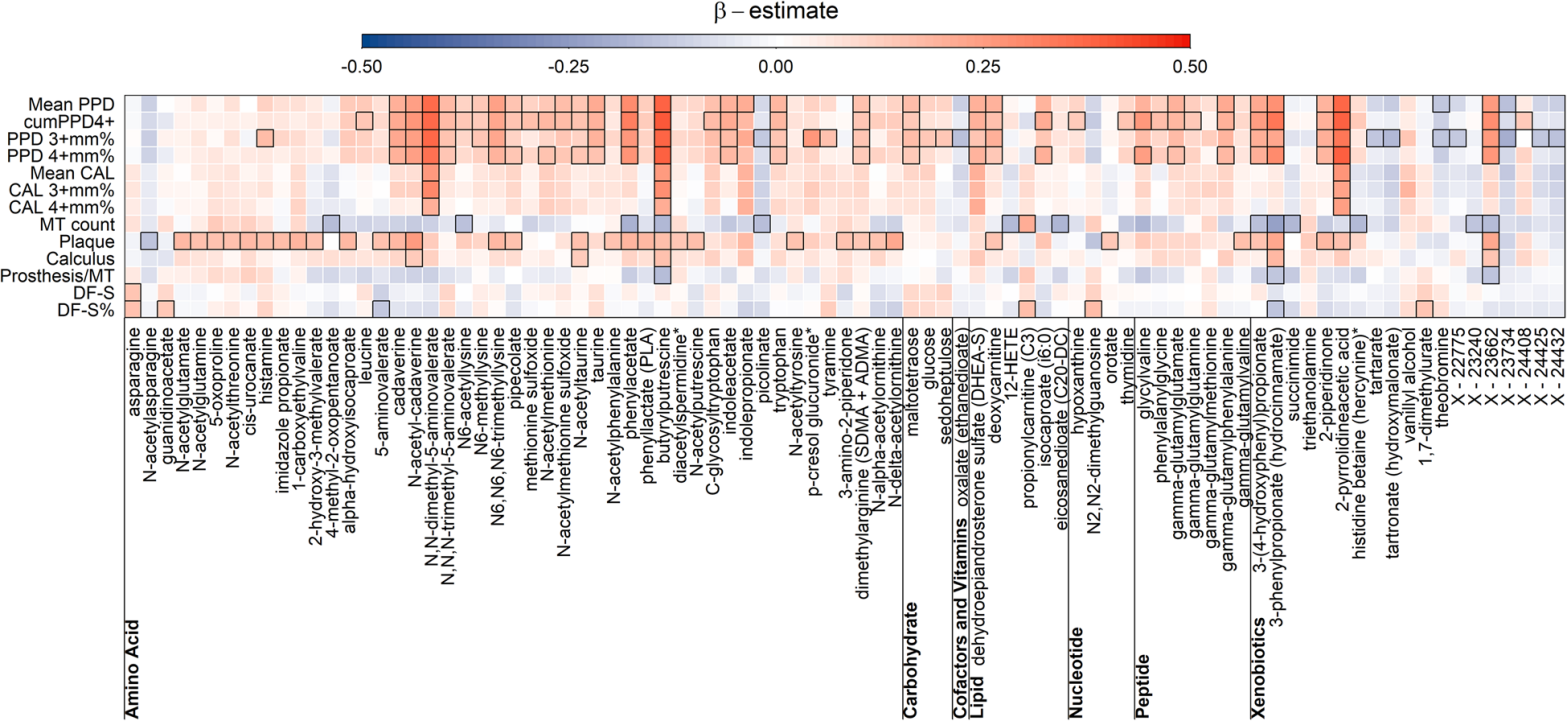
Heatmap of saliva metabolites significantly associated with at least 1 of the traits under investigation. Coloring reflects the direction and size of point estimates from linear regression models. Bordered fields mark combinations with FDR corrected *p* values <0.05. PPD, pocket probing depth; PPD 3+mm% and 4+mm%, percentages of sites with PPD ≥3 mm and ≥4 mm; cumPPD4+, cumulative PPD from pockets with PPD ≥4 mm; CAL, clinical attachment level; CAL 3+mm% and 4+mm%, percentages of sites with CAL ≥3 mm and ≥4 mm; MT, missing teeth; DF-S and DF-S%, number and percentage of decayed or filled surfaces

Firstly, to replicate previous findings [16], we cross-sectionally evaluated linear associations between the identically defined 13 dental exposure variables with saliva metabolite levels (outcome) adjusting for age (as restricted cubic splines with three knots), sex, BMI, smoking status, and diabetes mellitus (see Fig. 1). We compared effects estimates for 156 commonly detected metabolites with those published in [16] using Pearson correlation coefficients.

To test candidate metabolites for associations with a 5-year tooth loss, we used negative binomial regression models adjusted for the above-named confounders as well as respective baseline levels of the outcome and the follow-up time (as an offset variable). Only metabolites with ≥2 significant cross-sectional associations with PPD-, CAL-related variables, DFS, DFS%, or MT count were selected as exposure variables in longitudinal analyses. We derived incidence rate ratios (IRRs) and 95% confidence intervals.

We accounted for chance findings due to multiple testing by controlling the false discovery rate (FDR) at 5%, that is, approximately 5% of the metabolites presented as significant associations might be false-positive ones. This type of error control makes use of the fact that the distribution of *p* values of multiple unrelated statistical tests on the same cohort follows a uniform distribution if the null hypothesis is true. In other words, the *p* value can take each value between zero and one with an equal chance and an accumulation of small *p* values indicates the presence of true positive effects. The 5% threshold reflects this acceptance rate. Adjusted *p* values were referred to as *q* values.

We used Gaussian graphical modeling to derive a data-driven metabolic network based on 260 metabolites with less than 25% missing values and imputed remaining missing values using a Random Forest approach as implemented in the R package “missForest.”

For metabolites with significant associations in the longitudinal study part, we performed phenome-wide association (PheWAS) and genome-wide association studies (GWAS). The method is described in detail in the Supplementary Methods (Additional file 1 [31–40]).

All statistical analyses were done in R version 3.6.1 (www.r-project.org) and Stata/SE 14.2 (StataCorp, College Station, TX, USA).

## Results

A detailed description of the study population is given in Table S1 (Additional file 1). Briefly, participants were on average 53 years old, women and men were equally represented, 80.9% of whom were non-smokers at the time of the SHIP-2 examination, and 8.5% had known or diagnosed diabetes mellitus.

### Cross-sectional associations between oral variables and saliva metabolites

Each of the oral variables was significantly associated with saliva levels of at least one out of 84 metabolites (Fig. 1). Out of those, 51 metabolites have not been reported yet, which might be most likely attributable to the larger coverage of our metabolomics platform. PPD variables (e.g., cumPPD4+ associated with 36 metabolites) and plaque (36 hits) were the most prominent oral variables, whereas CAL variables associated with saliva levels of only a few metabolites (<10). Tooth count was in the middle of the spectrum. The metabolic profile for PPD variables largely overlapped, including strong positive associations with N-methylated amino acids, such as N,N-dimethyl-5-aminovalerate, catabolites of amino acids, such as phenylacetate, or metabolites of likely exogenous origin such as 2-pyrr. Some but not all of these associations were shared with plaque, which was additionally associated with members of the glutamate metabolism like N-actelyglutamate. We noted that most (168/198 = 85%) of the associations were positive, which might indicate generally higher metabolite levels in states of poor oral health, possibly driven by tissue degradation and enhanced microbial metabolism. Butyrylputrescine was associated with the highest number (*N* = 11) of oral variables.

**Fig. 2:**
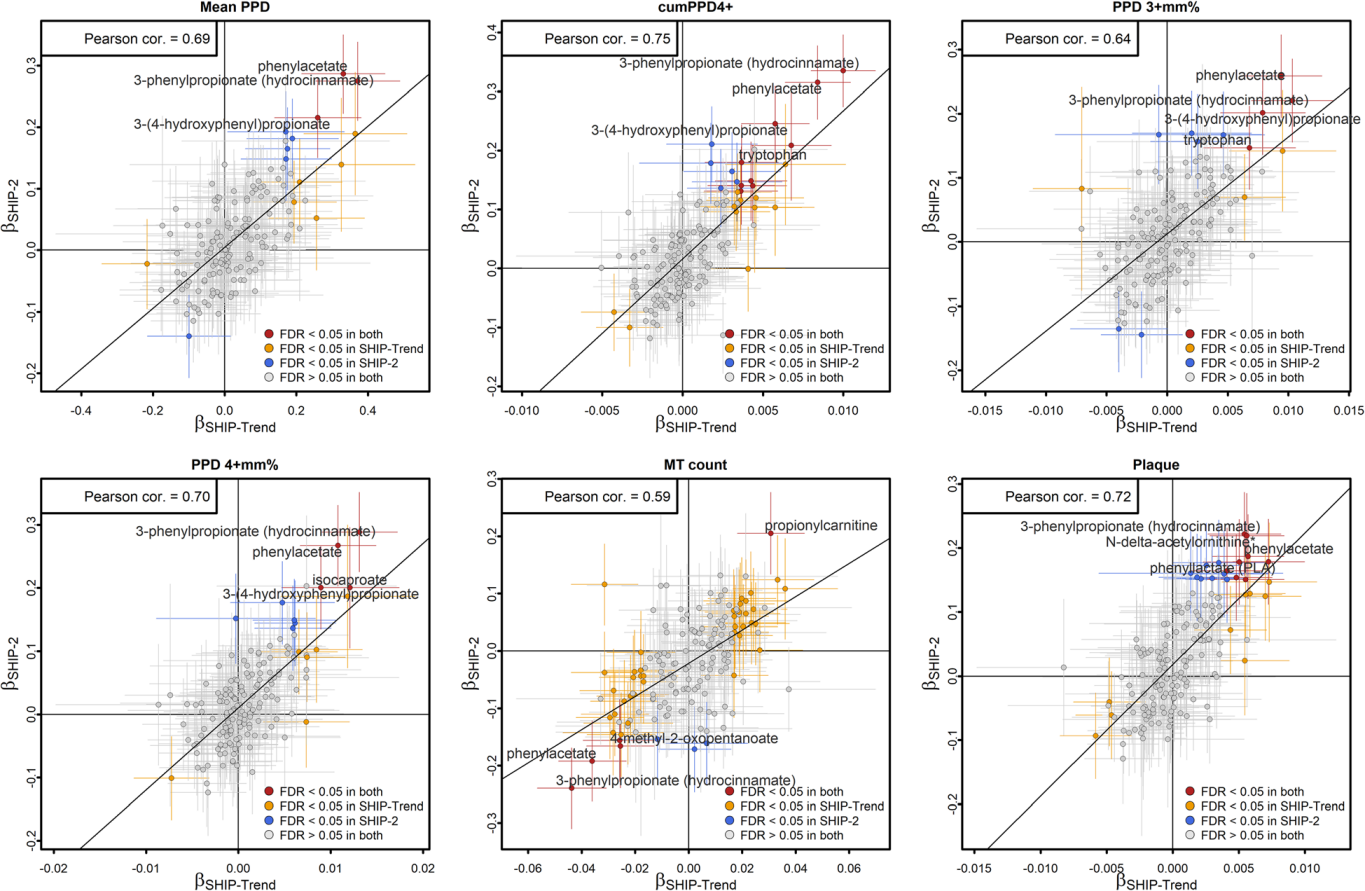
Point estimates (β) comparison from regression models for selected dental variables between SHIP-2 and SHIP-Trend [16]. The selected variables include the mean PPD, cumPPD4+, PPD 3+mm%, PPD 4+mm%, MT count, and plaque. PPD, pocket probing depth; PPD 3+mm% and 4+mm%, percentages of sites with PPD ≥3 mm and ≥4 mm; cumPPD4+, cumulative PPD from pockets with PPD ≥4 mm; MT, missing teeth

We observed high correlation coefficients between this study and our previous work when comparing effect estimates (Fig. 2, Additional file 1: Figure S1), most notably for oral variables that showed evidence for any association. In particular, we replicated our most prominent previous findings, namely 3-phenylpropionate, phenylacetate, and 3-(4-hydroxyphenyl)propionate with consistent effect directions.

### Association of saliva metabolites with a 5-year tooth loss

**Fig. 3:**
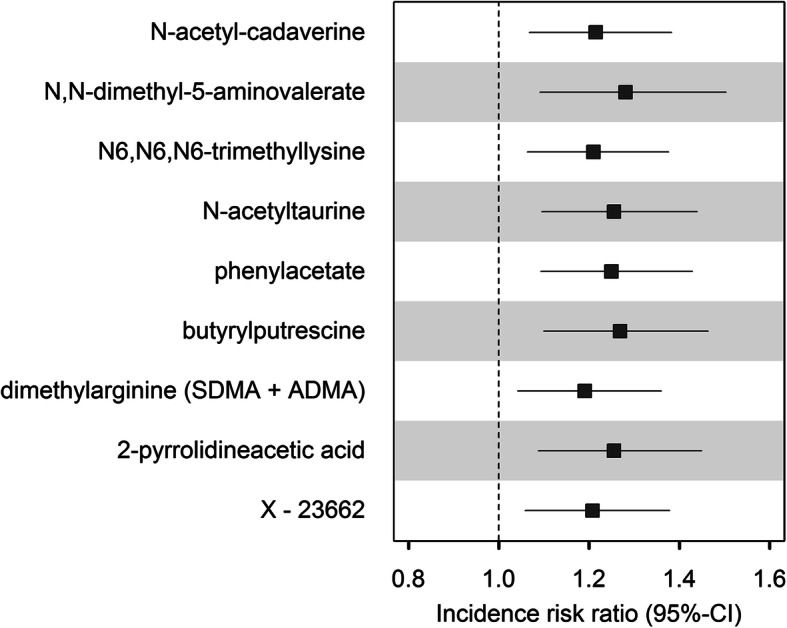
Forest plot of saliva metabolites significantly (*q *value<0.05) associated with a 5-year tooth loss. The graphic shows incidence rate ratios (with 95% confidence intervals) from negative binomial regression models for associations of saliva metabolites with a 5-year tooth loss using longitudinal data

A total of 34 metabolites were associated with two or more oral variables, which we used as candidates for longitudinal analyses to minimize the testing burden. Of the 34 candidate metabolites, nine were significantly associated with a 5-year tooth loss, even after adjustment for the baseline oral health status, with IRRs ranging between 1.19 and 1.28 (Fig. 3). The highest IRR value was estimated for N,N-dimethyl-5-aminovalerate (IRR 1.28 (95% CI 1.10, 1.49), *q* value=0.017). This indicated an additional effect of metabolite levels over and above what might have already been present at baseline.

### A saliva metabolite network

**Fig. 4:**
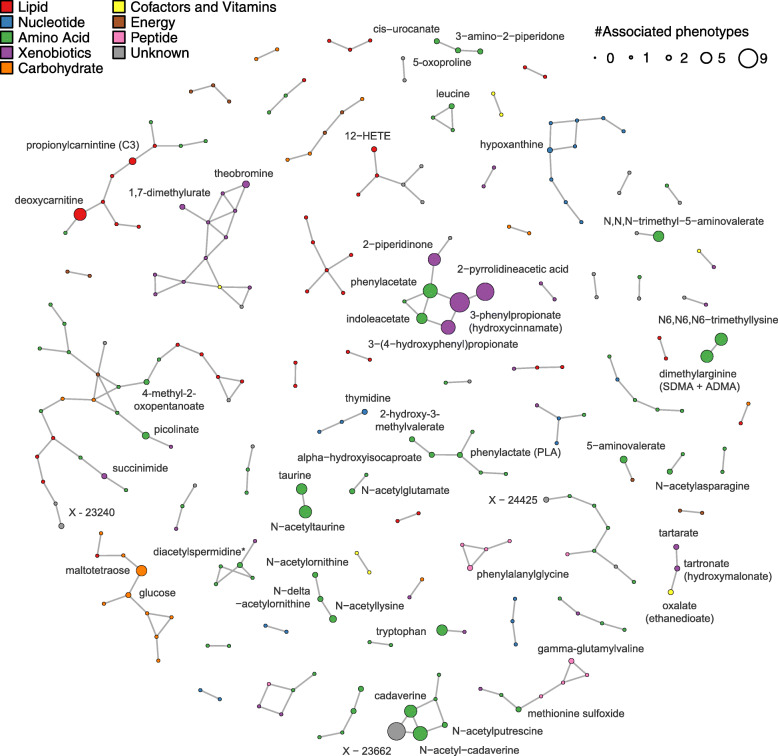
Partial correlation network of metabolites, having significant interactions with other metabolites. Metabolites are displayed as nodes and significant interactions are displayed as lines. Only metabolites with at least one association to a phenotype in the cross-sectional analysis were annotated. The size of a node corresponds to the number of associated phenotypes in cross-sectional analyses. The color markings classify the metabolites into different substance groups

To derive a sparse but informative network to link metabolites and hence group association patterns, we applied Gaussian graphical modeling and were able to map 260 metabolites linked by 215 edges (Fig. 4). Projecting the results from the cross-sectional analysis onto this network enabled us to identify groups of metabolites with less obvious connections compared to literature research. In particular, 2-pyrr belonged to a subnetwork with metabolites such as 3-phenylpropionate and phenylacetate.

### Phenome-wide association studies

To gain better insights into the metabolites showing significant associations with a 5-year tooth loss, we leveraged the deep phenotyping of the SHIP-2 cohort and tested associations with 59 different phenotypes (Additional file 1: Tables S2-S10). Smoking was significantly associated with saliva levels of 2-pyrr, X-23662, butyrylputrescine, N,N-dimethyl-5-aminovalerate, cadaverine, and N-acetyltaurine (see Table 1). Characteristics related to cardio-metabolic disorders were significantly associated with saliva levels of X-23662, cadaverine, N6,N6,N6-trimethyllysine, and N-acetyltaurine. Inflammatory markers were significantly associated with saliva levels of 2-pyrr, X-23662, butyrylputrescine, cadaverine, N6,N6,N6-trimethyllysine, and dimethylarginine. Finally, obesity was significantly associated with saliva levels of X-23662, cadaverine, N6,N6,N6-trimethyllysine, phenylacetate, and dimethylarginine.

**Table 1:** Overview on PheWAS results for nine metabolites significantly related to a 5-year tooth loss

	PheWAS variable	2-Pyrrolidineacetic acid	X-23662	Butyrylputrescine	N,N-dimethyl-5-aminovalerate	N-acetyl-cadaverine	N6,N6,N6-trimethyllysine	N-acetyltaurine	Phenylacetate	Dimethylarginine (SDMA + ADMA)
Smoking	Smoking (current)	+ 0.0001	+ 0.031	+ 0.00013	+ 0.0011	+ 0.047		- 0.020		
Cardio-metabolic disorders	Known or diagnosed diabetes mellitus		+ 0.030							
	Known diabetes mellitus		+ 0.023							
	Diagnosed high blood pressure		+ 0.026							
	Systolic blood pressure (mmHg)		+ 0.026			+ 0.034	+ 0.023			
	Triglycerides (total) (mmol/l)							- 0.020		
Inflammation	Fibrinogen acc. to Clauss (g/l)			+ 0.026			+ 0.023			+ 0.019
	Leukocytes (Gpt/l)	+ 0.03	+ 0.0003	+ 0.00044		+ 0.000004	+ 0.021			+ 0.002
	Mean corpuscular haemoglobin (mmol/l)		- 0.023			- 0.015				
	Red cell distribution width (%)									+ 0.019
Obesity	Weight (kg)		+ 0.003			+ 0.002	+ 0.015			
	Waist circumference (cm)		+ 0.0003			+ 0.0006	+ 0.023		+ 0.032	+ 0.019
	Hip circumference (cm)		+ 0.0067			+ 0.005	+ 0.032			+ 0.047
Others	Bad breath			+ 0.033						
	Sodium (mmol/l)			+ 0.029						
	Serum amylase (μkatal/l)						- 0.023			

Only associations with *q* < 0.05 are listed. Plus sign: Beta (from linear regression models) >0; minus sign: Beta<0; Benjamini-Hochberg-adjusted *p* values are given (referred to as *q* value)

### Genome-wide association studies

We performed genome-wide association studies to identify genetic determinants of two of our most promising candidate metabolites 2-pyrr and butyrylputrescine motivated by earlier studies with strong effects even at a small or moderate scale [41]. However, we obtained no signal passing the genome-wide significance threshold (*p* < 5e−8) in a standard GWAS analysis (Additional file 1: Supplementary Methods and Results, Table S11 and Figures S2 and S3).

## Discussion

Periodontitis is a common chronic condition associated with a large comorbidity burden, and we investigated molecular profiles of saliva samples to identify pathways of periodontitis and tooth loss progression in a large population-based sample. We identified a broad range of periodontitis-associated metabolites indicating tissue destruction, dysbiosis of the oral microbiome, and possibly cell proliferation in cross-sectional analysis replicating and augmenting previous work [16]. Non-invasive biomarkers identifying people at highest risk can spare time for both, the patient and the dentist, because periodontitis is a silent disease, often overlooked and undertreated in dental offices and can only be accurately diagnosed by cumbersome clinical examinations with a certain discomfort.

We next showed that molecular profiles of periodontitis, representing multiple biological mechanisms, partially translate into prospective associations, that is, saliva levels at baseline being strongly associated with tooth loss after 5 years of follow-up. While tooth loss can be caused by multiple factors, including caries, trauma, and prosthetic treatment decisions, periodontitis is the most important contributor among middle-aged and older individuals and the convergence of metabolites associated with periodontitis at baseline on tooth loss at follow-up adds a novel biological layer, including severe periodontal breakdown, which triggered the dentist to extract these teeth. At baseline, subjects without tooth loss had a mean CAL of 2.28±1.17 mm, while subjects with tooth loss had a mean CAL of 3.13±1.44 mm (Additional file 1: Table S1). On tooth level, retained teeth had a maximum CAL of 3.22±1.74 mm, whereas extracted teeth had a maximum CAL of 6.07±3.06 mm. Thus, the periodontal breakdown was closely related to a 5-year tooth loss in SHIP.

We showed that nine metabolites, including the yet unreported 2-pyrr and butyrylputrescine, as well as previously reported ones such as phenylacetate, associated with a 5-year tooth loss over and above oral health status at baseline pointing towards the opportunity to use simple non-invasive saliva sampling to identify people at risk for tooth loss. Only one (phenylacetate) of the nine identified metabolites was also associated with oral health status in the cross-sectional analysis, clearly emphasizing the gain using the state-of-the-art metabolomics techniques for biomarker discovery. In accordance with previous studies, the identified molecular signatures for tooth loss point to surrogates of bacterial metabolism, cell proliferation, and host defense mechanisms (Additional file 1: Table S12). These data support the assumption that these periodontal metabolites predict tooth loss.

We chose a 5-year tooth loss as the primary outcome in longitudinal analysis, as it represents the most severe end of the spectrum of periodontitis. To our knowledge, we are the first to successfully associate baseline metabolite levels in saliva with a 5-year tooth loss, thereby opening also new perspectives for tooth loss prediction.

### 2-Pyrrolidineacetic acid

Saliva levels of 2-pyrr were strongly associated with tooth loss. Biomedical literature about this metabolite, however, is sparse. One potential biological source of 2-pyrr is the tobacco plant [42] and our PheWAS approach highlighted smoking as one of the very few health characteristics associated with 2-pyrr (see Additional file 1: Figure S4), and it is well known that smoking increases periodontal disease progression [43]. Besides 2-pyrr being an exogenous smoking metabolite, smoking shifts the subgingival microbiome towards a pathogen enriched community [44]. This shift may favor bacterial species that can produce 2-pyrr or related catabolites. The high detection frequency of 2-pyrr across all participants makes it, however, less likely that 2-pyrr is solely of exogenous origin or due to smoking-induced microbiome shift. A hypothesis further supported by the persistence of the association in statistical models controlling for smoking status. The missing association with other major cardiometabolic and anthropometric health variables in our PheWAS pinpoints to the local origin of 2-pyrr, which is supported by the metabolic network, in which 2-pyrr was in close proximity to previously described bacterial degradation products such as 3-phenylpropionate, phenylacetate, or 3-(4-hydroxyphenyl)propionate (Fig. 4) [16, 45]. Another potential local source of 2-pyrr could be collagen turnover in the periodontium, since pyrrolidine residues contribute to the stability of collagen [46] and increased collagen turnover has been suggested as a predictive marker of alveolar bone loss [47].

### Butyrylputrescine, N-acetylcadaverine, and N,N-dimethyl-5-aminovalerate

Butyrylputrescine and N-acetylcadaverine, which are both polyamine derivatives, were among the most consistently associated metabolites in our study and our PheWAS results (Table 1) confirmed the association of butyrylputrescine with bad breath (using a “Sulphide Monitor”) [48], a typical symptom of periodontitis. Polyamines contribute to various eukaryotic and prokaryotic cellular processes, and their levels are tightly controlled by multiple factors [49, 50], as both the absence and excessive accumulation of polyamines lead to cell death [51]. The fact that higher levels of N-acetylcadaverine associated with periodontitis and ensuing tooth loss might therefore indicate a local excess of polyamines in the periodontal tissues [18, 45, 52]. The primary source of polyamine excess, however, remains to be established, but our PheWAS approach implicated systemic inflammatory host factors to be associated with saliva levels of polyamines (Table 1), which may be caused by a spillover of inflammatory markers into the circulation. One might speculate that, again, oral microbiota contribute to polyamine excess by metabolizing periodontal host amino acids such as ornithine and lysine [53, 54]. Further, 5-aminovalerate is a metabolite produced by the catabolism of cadaverine [55] and its increased occurrence induces cellular excess of cadaverine. Altogether, the increased bacterial load associated with periodontitis might lead to increased polyamine levels reflected by the mutual increase of modified catabolites, which may have a longer half-life in the oral metabolome.

### Dimethylarginine and N6,N6,N6-trimethyllysine

Various oral bacteria rely on the breakdown of host dipeptides as an energy resource and the documented association of dimethylarginine and N6,N6,N6-trimethyllysine with a 5-year tooth loss might well fit into this pattern. Briefly, periodontal tissue breakdown is carried out by activated proteases of host and bacteria, resulting in higher levels of dipeptides [17, 56], free amino acids, and amino acid metabolites [16, 18]. The ability of bacteria to produce proteolytic enzymes constitutes a survival strategy [57, 58]. Dimethylarginine and N6,N6,N6-trimethyllysine are arginine and lysine derivatives, and they might be linked to *Porphyromonas (P.) gingivalis*. *P. gingivalis* is considered a central periodontal pathogen, which shifts a healthy into a dysbiotic subgingival microbiome, initiating inflammatory breakdown [12]. Arginine-gingipain and lysine-gingipain, which are virulence factors of *P. gingivalis*, cleave protein substrates after arginine and lysine residues in oligopeptides [59, 60].

### Phenylacetate

We replicated our previous observation of a strong and consistent association between saliva levels of phenylacetate and oral variables and further showed that saliva levels were associated with a 5-year tooth loss. While we speculated previously that 3-phenylacetate, similar to 3-phenylpropionate and 3-(4-hydroxyphenyl)propionate, likely originates from microbial metabolism, the current study allowed us to expand on this. The derived metabolic network puts those metabolites in close proximity to 2-pyrr, which might point towards a common community of bacteria contributing to the saliva pool of all of these metabolites. Further, our PheWAS approach identified waist circumference (which is a host factor) positively associated with phenylacetate, which led us to hypothesize on a systemic origin of phenylacetate. This metabolite has been measured in body fluids other than saliva, including blood and urine [61–63], and (passive) exchange processes between blood and saliva might explain a connection between both. However, higher plasma levels of phenylacetate have been associated with chronic renal failure [64], arterial vascular properties in patients with end-stage chronic kidney disease [61], and high fat diet-induced initiation of insulin resistance in mice [65].

### N-acetyltaurine

The association between saliva levels of N-acetyltaurine and a 5-year tooth loss is puzzling, since little is known about this compound in general. It may relate to the antioxidative properties of its closely related compound taurine [66, 67], which might have a role in the host response releasing reactive oxygen species to fight bacteria. If the imbalance between reactive oxygen species and the antioxidant defense continues in favor of free radicals, the antioxidant capacity is exhausted, which contributes to periodontal tissue destruction [68]. However, N-acetyltaurine might also be host derived since saliva levels were associated with host characteristics, such as smoking and serum triglyceride levels (see Table 1), and previous studies have already linked this metabolite to exogenous health-modifying circumstances, including intake of alcohol and endurance sports [69–72].

### Strengths and limitations of our study

Major strengths of our analyses are the longitudinal design, large sample size, replication of cross-sectional findings [16], and a detailed assessment of the oral health status. However, our study was explorative and not designed to establish causality.

### Clinical implications

Periodontitis is often overlooked [73] and undertreated, and there is no accurate biomarker to indicate the presence of periodontitis and/or to monitor periodontal stability after active treatment. In our cross-sectional analysis, 2-pyrr and N6,N6,N6-trimethyllysine were not significantly associated with supragingival plaque, which causes gingivitis, but with phenotypes of periodontitis. This finding indicates that at least these two biomarkers are exclusively increased during periodontal breakdown and thus might be highly sensitive for periodontitis. Further, prospective studies are needed to quantify the sensitivity, specificity, and diagnostic accuracy of such biomarkers or combinations thereof.

Preventive dental care in which consumers can evaluate their periodontal health and communicate with the dental team on an ongoing or as-needed basis may reduce health care cost and offer new possibilities to deliver care. A point-of-care (POC) device, based on non-invasive biomarkers, such as identified in the present study, may open a new avenue for such periodontal home care. If patients were able to screen themselves for periodontitis, they could initiate self-treatment, because incipient and moderate periodontitis can be halted or at least retarded by improved oral home care measures [74].

To improve prediction, many attempts were made and some commercial kits based MMP-8 as the product of the host response or on putative periodontal pathogens were launched, but neither of these biomarkers was sensitive and specific enough and no breakthrough was achieved for monitoring periodontal stability [75]. Furthermore, periodontal progression is often very slow and maintenance sessions could be extended [76], if home monitoring by these biomarkers would be feasible. Future treatment studies have to find out if our detected biomarkers add diagnostic benefit to periodontal care.

If a combination of biomarkers was predictive for future tooth loss, the periodontal community would have a tool to deliver a tailored periodontal therapy. A large multicenter study showed that hitherto used clinical variables were insufficient to predict tooth loss [77]. The 2017 World Workshop on the classification of periodontal conditions suggested that the extent of tooth loss has to be a part of staging periodontal disease severity, but periodontitis case definitions should extend beyond phenotypic disease symptom description and include biological features, which may help to adopt a more precise periodontitis management in the future, because not all patients respond equally good to standard therapeutic measures and some patients are more prone to tooth loss than others. It provided a diagnostic framework which calls for the inclusion of biomarkers for prognosis [78].

## Conclusions

We identified a distinct profile of saliva metabolites associated with tooth loss. Specifically, 2-pyrr and butyrylputrescine were strongly associated with a 5-year tooth loss over and above current oral status and might hence have potential as biomarkers for screening purposes to identify periodontally diseased subjects (diagnostic), but also to identify subjects at risk for more tooth loss (prediction). The metabolic profile seemed to be enriched for markers of a dysbiotic oral microbiome and the respective host response. The ability of identified candidate metabolites to enable effective screening or prediction has to be established in appropriate settings. However, it should be noticed that tooth loss is not an outcome that is specific to periodontitis. Thus, the saliva metabolites associated with tooth loss may also be linked with other non-periodontal mechanisms. We further note that replication of the results in cohorts of different age and ethnic distribution, and health conditions is warranted, as is the integration of oral microbiome data to improve our understanding of how host and microbial metabolism is interlinked and possibly drives periodontitis progression.

## Supplementary Information

**Additional file 1.** Title of data: Detailed information about baseline characteristics of study participants, the in-vitro test, the phenome-wide association study, and the genome-wide association study. Description of data: We provide detailed information about baseline characteristics of study participants, the phenome-wide association study (for 2-pyrrolidineacetic acid, X-23662, butyrylputrescine, N,N-dimethyl-5-aminovalerate, N-acetylcadaverine, N6,N6,N6-trimethyllysine, N-acetyltaurine, phenylacetate, dimethylarginine (SDMA + ADMA)), and the genome-wide association study (for 2-pyrr and butyrylputrescine). Information is split into the Supplementary Methods, the Supplementary Results, the Tables S1-S12, and the Figures S1-S4.

## Data Availability

Data from SHIP are available after data application and signature of a data transfer agreement. The data dictionary and the online application form are available at: fvcm.med.uni-greifswald.de/dd_service/data_use_intro.php. Involving a local collaborative partner to facilitate the application process is recommended.

## Abbreviations

BMI: Body mass index
CAL: Clinical attachment level
cumPPD4+: Cumulative PPD from pockets with PPD ≥4 mm
FDR: False discovery rate
GWAS: Genome-wide association study
PheWAS: Phenome-wide association study
HbA1c: Glycated hemoglobin
IRR: Incidence rate ratio
MS/MS: Mass spectrometry/mass spectrometry
MT count: Total number of missing teeth
PCP-11: Periodontal clinical probe 11
PPD: Pocket probing depth
PPD 3+mm%: Percentage of sites with PPD ≥3 mm
PPD 4+mm%: Percentage of sites with PPD ≥4 mm
SHIP: Study of Health in Pomerania
ULPLC-MS/MS: Ultra-high-performance liquid-chromatography and tandem mass spectrometry
WBC: White blood cell count
2-pyrr: 2-Pyrrolidineacetic acid
