# Supplementary material for: Salivary metabolites associated with a 5-year tooth loss identified in a population-based setting
Source: BMC Med. 2021 Jul 14;19:161. doi: 10.1186/s12916-021-02035-z (PMC8278731; doi:10.1186/s12916-021-02035-z)
Supplement: Supplementary file 1 — Additional file 1. Title of data: Detailed information about baseline characteristics of study participants, the in-vitro test, the phenome-wide association study, and the genome-wide association study. Description of data: We provide detailed information about baseline characteristics of study participants, the phenome-wide association study (for 2-pyrrolidineacetic acid, X-23662, butyrylputrescine, N,N-dimethyl-5-aminovalerate, N-acetylcadaverine, N6,N6,N6-trimethyllysine, N-acetyltaurine, phenylacetate, dimethylarginine (SDMA + ADMA)), and the genome-wide association study (for 2-pyrr and butyrylputrescine). Information is split into the Supplementary Methods, the Supplementary Results, the Tables S1-S12, and the Figures S1-S4. [file 12916_2021_2035_MOESM1_ESM.docx]

**Supplementary Methods**

*Phenome-wide association study*

In contrast to the widely applied genome-wide association studies, phenome-wide analyses (PheWAS) have been introduced with the aim to identify associations of multiple phenotypes with a specific genetic variant (69). In the context of this analysis PheWAS has been used to study associations with salivary metabolites, using a function in Stata, written by COS. Associations with in total 68 variables of interest have been analysed. Depending in the type of the phenotype one of the following regression types is chosen by the function: logistic regression, linear regression, ordinal regression, count regression, zero-inflated negative binomial regression.

*Genome-wide association study*

SHIP participants were genotyped using the Affymetrix Genome-Wide Human SNP Array 6.0 (Santa Clara, CA, USA) genotyping array in accordance with the manufacturer’s standard protocol. We performed whole genome imputation of the genotypes on the Michigan Imputation Server (70) using the HRC (r1.1) reference panel. Details on genotyping, quality control and imputation are provided in Supplementary Table 11.

Single variant association analyses on all variants were performed using snptest v2.5.2 (71) considering an additive genetic model. We performed multiple linear regression analysis, adjusted for age at blood taking, sex, smoking status (current vs former vs non-smokers) and the first ten principal components to correct for population stratification.

**Supplementary Results**

*Genome-wide association study*

Although our previous work revealed strong genetic associations with metabolites in saliva in a similarly sized cohort (31), we were not able to identify genetic variants passing the genome-wide significance threshold in the current study (see Supplementary Figures 3 and 4). We note that a meta-analysis with previous studies was not possible due to missing coverage of our candidate metabolites in the Twins-UK sample.

**Supplementary Tables**

**Supplementary Table 1. Baseline characteristics of study participants (at SHIP-2) included in cross-sectional and longitudinal analyses.**

|  |  |  | Number of extracted teeth between  SHIP-2 and SHIP-3 | |
| --- | --- | --- | --- | --- |
| Variable | N | Total sample | 0 | >0 |
| Age, years | 938 | 52.6 ± 11.8 | 50.9 ± 11.7 | 56.9 ± 11.2 |
| Sex |  |  |  |  |
| Male | 938 | 474 (50.5%) | 332 (49.5%) | 142 (53.2%) |
| Female |  | 464 (49.5%) | 339 (50.5%) | 125 (46.8%) |
| Smoking status | 938 |  |  |  |
| Never smoker |  | 359 (38.3%) | 280 (41.7%) | 79 (29.6%) |
| Ex-smoker |  | 400 (42.6%) | 285 (42.5%) | 115 (43.1%) |
| Current smoker |  | 179 (19.1%) | 106 (15.8%) | 73 (27.3%) |
| Body mass index, kg/m^2^ | 938 | 27.7 ± 4.6 | 27.5 ± 4.6 | 28.4 ± 4.6 |
| Known or diagnosed diabetes mellitus, yes | 938 | 80 (8.5%) | 49 (7.3%) | 31 (11.6%) |
| ***Dental variables*** | | | |  |
| Mean PPD, mm | 938 | 2.53 ± 0.47  2.46 (2.23; 2.75) | 2.47 ± 0.39  2.43 (2.21; 2.70) | 2.70 ± 0.58  2.60 (2.32; 2.97) |
| Cumulative PPD, mm | 938 | 19.2 ± 24.5  10 (0; 28) | 16.8 ± 21.7  8 (0; 25) | 25.3 ± 29.6  18 (4; 33) |
| Percentage of sites with PPD ≥3 mm, % | 938 | 48.3 ± 19.9  47.6 (34.6; 61.5) | 46.0 ± 19.1  44.6 (32.5; 60.0) | 54.1 ± 20.7  51.8 (40.0; 71.4) |
| Percentage of sites with PPD ≥4 mm, % | 938 | 10.2 ± 12.5  5.8 (0; 15.4) | 8.4 ± 10.4  4.2 (0; 12.5) | 14.8 ± 15.7  10.7 (2.5; 20.8) |
| Mean CAL, mm | 937 | 2.52 ± 1.31  2.38 (1.54; 3.21) | 2.28 ± 1.17  2.15 (1.44; 2.92) | 3.13 ± 1.44  2.87 (2.13; 4.04) |
| Percentage of sites with CAL ≥3 mm, % | 937 | 45.1 ± 32.4  42.5 (15.0; 72.2) | 39.7 ± 31.1  36 (11.4; 64.6) | 58.8 ± 31.6  62.5 (35.0; 87.5) |
| Percentage of sites with CAL ≥4 mm, % | 937 | 23.3 ± 27.3  12.8 (1.8; 35.7) | 18.6 ± 23.8  8.8 (0; 27.5) | 35.3 ± 31.6  26.1 (7.1; 60.7) |
| Number of missing teeth | 938 | 4.8 ± 4.9  3 (1; 7) | 4.1 ± 4.6  3 (1; 5) | 6.6 ± 5.3  6 (3; 9) |
| Number of extracted teeth | 938 | 0.51 ± 1.12  0 (0; 1) | - | 1.81 ± 1.43  1 (1; 2) |
| Percentage of sites with plaque, % | 938 | 18.9 ± 22.1  12.5 (0; 29.2) | 17.4 ± 21.4  8.3 (0; 25) | 22.6 ± 23.5  16.7 (4.2; 33.3) |
| Percentage of sites with calculus, % | 938 | 6.9 ± 9.5  4.2 (0; 8.7) | 6.2 ± 9.2  4.2 (0; 8.3) | 8.4 ± 10.0  5 (0; 12.5) |
| Prosthesis; number of missing teeth | 938 |  |  |  |
| No; 0-8 |  | 740 (78.9%) | 571 (85.1%) | 169 (63.3%) |
| No; 9-27 |  | 32 (3.4%) | 16 (2.4%) | 16 (6.0%) |
| Yes; 0-8 |  | 38 (4.1%) | 15 (2.2%) | 23 (8.6%) |
| Yes; 9-27 |  | 128 (13.7%) | 69 (10.3%) | 59 (22.1%) |
| DF-S | 938 | 18.5 ± 9.2  18 (12; 25) | 18.2 ± 9.0  18 (12; 24) | 19.1 ± 9.7  18 (12; 26) |
| DF-S%, % | 938 | 36.3 ± 18.6  35.2 (22.2; 49.0) | 34.7 ± 18.0  33.3 (21.9; 46.0) | 40.3 ± 19.5  40.9 (25.9; 53.7) |
| ***Saliva metabolites*** |  |  |  |  |
| N-acetyl-cadaverine | 931 | -0.006 ± 0.997 | -0.057 ± 0.970 | 0.119 ± 1.053 |
| N,N-dimethyl-5-aminovalerate | 567 | -0.017 ± 0.998 | -0.080 ± 0.949 | 0.123 ± 1.090 |
| N6,N6,N6-trimethyllysine | 928 | -0.002 ± 1.006 | -0.051 ± 0.990 | 0.121 ± 1.038 |
| N-acetyltaurine | 916 | 0.006 ± 0.998 | -0.038 ± 1.017 | 0.117 ± 0.941 |
| Phenylacetate | 933 | 0.008 ± 0.994 | -0.043 ± 0.969 | 0.134 ± 1.047 |
| Dimethylarginine | 910 | -0.008 ± 1.000 | -0.077 ± 1.002 | 0.163 ± 0.977 |
| 2-pyrrolidineacetic acid | 722 | -0.015 ± 0.992 | -0.095 ± 0.965 | 0.170 ± 1.032 |
| Butyrylputrescine | 891 | 0.000 ± 0.999 | -0.051 ± 0.968 | 0.130 ± 1.066 |
| X-23662* | 721 | -0.005 ± 0.987 | -0.059 ± 0.959 | 0.119 ± 1.041 |

Data are presented as mean ± standard deviation, median (25% quantile; 75% quantile), or numbers (percentages). Abbreviations: N, number of subjects with non-missing data in the total sample; DF-S, number of decayed or filled surfaces; DF-S%, percentage of decayed or filled surfaces; PPD, periodontal probing depth; CAL, clinical attachment level; MT, missing teeth. *unknown metabolite

**Supplementary Table 2. Linear regression model results evaluating associations of various variables with saliva levels of 2-pyrrolidineacetic acid.**

| Variable | Level | N | B | SE | P value | q value* |
| --- | --- | --- | --- | --- | --- | --- |
| ***Medical diseases*** |  |  |  |  |  |  |
| **Smoking (ref. never smoker)** | **Ex-smoker** | **756** | **0.036** | **0.082** | **0.67** |  |
|  | **Current smoker** |  | **0.491** | **0.101** | **<0.001** | **0.0001** |
| Known or diagnosed diabetes mellitus | no/yes | 723 | -0.108 | 0.132 | 0.41 | 0.77 |
| Known diabetes mellitus | no/yes | 720 | -0.028 | 0.148 | 0.85 | 0.95 |
| Self-reported general state of health (Excellent or very good, ref.) | good | 757 | -0.170 | 0.086 | 0.05 |  |
|  | less good or bad |  | -0.269 | 0.141 | 0.06 | 0.57 |
| Has a raised or high blood pressure (hypertension) been determined medically since the last SHIP-study? | no/yes | 755 | 0.094 | 0.078 | 0.23 | 0.59 |
| Heart rate (bpm) | continuous | 755 | -0.001 | 0.003 | 0.88 | 0.96 |
| Diastolic blood pressure (mmHg) | continuous | 753 | 0.004 | 0.004 | 0.25 | 0.59 |
| Systolic blood pressure (mmHg) | continuous | 751 | 0.003 | 0.002 | 0.12 | 0.57 |
| Do you often suffer from a bad taste in your mouth? | no/yes | 756 | 0.99 | 0.101 | 0.33 | 0.68 |
| Do you often suffer from a bad breath? | no/yes | 708 | 0.153 | 0.107 | 0.15 | 0.57 |
| Did you suffer more than once from pancreatitis? | no/yes | 750 | 0.043 | 0.409 | 0.92 | 0.96 |
| Have you had one of the following medical problems (diseases) within the last 12 months? |  |  |  |  |  |  |
| Varicose veins, thrombosis, phlebitis | no/yes | 755 | -0.118 | 0.138 | 0.39 | 0.76 |
| Inflammatory skin diseases (e.g. acne) | no/yes | 752 | -0.199 | 0.132 | 0.13 | 0.57 |
| degeneration of the joints, e.g. arthrosis of the hip, knee, shoulder, or foot joints | no/yes | 753 | -0.225 | 0.091 | 0.01 | 0.23 |
| inflammatory joint diseases, e.g. chronic polyarthritis | no/yes | 748 | -0.228 | 0.200 | 0.25 | 0.59 |
| osteoporosis, i.e. reduced bone density | no/yes | 749 | -0.279 | 0.206 | 0.18 | 0.57 |
| gastritis | no/yes | 754 | -0.109 | 0.196 | 0.58 | 0.82 |
| hepatitis | no/yes |  | -0.273 | 0.578 | 0.64 | 0.84 |
| fatty liver | no/yes | 753 | 0.075 | 0.144 | 0.60 | 0.83 |
| increased level of blood lipids (cholesterol, triglycerides) | no/yes | 750 | -0.048 | 0.092 | 0.61 | 0.83 |
| gout or increased uric acid | no/yes | 750 | 0.008 | 0.145 | 0.96 | 0.96 |
| chronic bronchitis, i.e. coughing phlegm in the morning on most days, at least 3 months within the last 2 years? | no/yes | 756 | -0.248 | 0.199 | 0.21 | 0.57 |
| Have you ever had cancer? | no/yes | 755 | -0.223 | 0.167 | 0.18 | 0.57 |
| Do you suffer from a medically determined chronic lung disease? | no/yes | 756 | -0.027 | 0.216 | 0.90 | 0.96 |
| Do you suffer from psoriasis? | no/yes | 754 | 0.109 | 0.173 | 0.53 | 0.82 |
| Have you suffered from gastritis within the last six months? | no/yes | 754 | 0.128 | 0.216 | 0.55 | 0.82 |
| Have you ever had a stomach (gastric) ulcer? | no/yes | 754 | -0.145 | 0.132 | 0.27 | 0.61 |
| ***Laboratory variables*** |  |  |  |  |  |  |
| Sodium (mmol/l) | continuous | 757 | 0.029 | 0.016 | 0.07 | 0.57 |
| Potassium (mmol/l) | continuous | 757 | 0.122 | 0.098 | 0.21 | 0.57 |
| Serum creatinine (µmol/l) | continuous | 757 | -0.002 | 0.003 | 0.37 | 0.74 |
| Serum uric acid (µmol/l) | continuous | 757 | 0.0001 | 0.001 | 0.81 | 0.95 |
| Serum glucose (mmol/l) | continuous | 757 | 0.005 | 0.026 | 0.83 | 0.95 |
| Cystatin c (mg/l) | continuous | 756 | -0.473 | 0.310 | 0.13 | 0.57 |
| Serum calcium, total (mmol/l) | continuous | 757 | 0.439 | 0.377 | 0.24 | 0.59 |
| Magnesium (mmol/l) | continuous | 757 | -0.159 | 0.403 | 0.69 | 0.87 |
| QUICK/Thromboplastin time (%) | continuous | 735 | -0.0001 | 0.002 | 0.95 | 0.96 |
| Thromboplastin time international normalized ratio | continuous | 753 | 0.044 | 0.160 | 0.78 | 0.93 |
| Partial thromboplastin time (s) | continuous | 754 | -0.008 | 0.012 | 0.51 | 0.82 |
| Fibrinogen acc. to Clauss (g/l) | continuous | 753 | 0.071 | 0.054 | 0.19 | 0.57 |
| Haemoglobin A1c (%) | continuous | 757 | -0.019 | 0.049 | 0.70 | 0.87 |
| Creatinine kinase, total (µkatal/l) | continuous | 757 | -0.002 | 0.031 | 0.95 | 0.96 |
| Lactate dehydrogenase (µkatal/l) | continuous | 757 | 0.005 | 0.086 | 0.95 | 0.96 |
| **Leukocytes (Gpt/l)** | **continuous** | **756** | **0.068** | **0.020** | **0.001** | **0.03** |
| Erythrocytes (tpt/l) | continuous | 756 | -0.060 | 0.102 | 0.56 | 0.82 |
| Haemoglobin (mmol/l) | continuous | 756 | -0.012 | 0.057 | 0.84 | 0.95 |
| Haematocrit | continuous | 756 | 0.705 | 1.264 | 0.58 | 0.82 |
| Mean corpuscular volume (fl) | continuous | 756 | 0.017 | 0.009 | 0.07 | 0.57 |
| Mean corpuscular haemoglobin (fmol) | continuous | 756 | 0.178 | 0.378 | 0.64 | 0.84 |
| Mean corpuscular haemoglobin concentration (mmol/l) | continuous | 756 | -0.139 | 0.062 | 0.03 | 0.51 |
| Red cell distribution width (%) | continuous | 756 | 0.059 | 0.044 | 0.18 | 0.57 |
| Thrombocytes (gpt/l) | continuous | 756 | -0.0003 | 0.001 | 0.69 | 0.87 |
| Mean platelet volume (fl) | continuous | 756 | 0.048 | 0.031 | 0.13 | 0.57 |
| Triglycerides (total) (mmol/l) | continuous | 757 | 0.042 | 0.028 | 0.14 | 0.57 |
| Cholesterol (total) (mmol/l) | continuous | 755 | 0.028 | 0.034 | 0.42 | 0.77 |
| LDL-cholesterol (mmol/l) | continuous | 757 | 0.027 | 0.039 | 0.50 | 0.82 |
| HDL-cholesterol (mmol/l) | continuous | 757 | -0.139 | 0.105 | 0.19 | 0.57 |
| Alanine aminotransferase (µkatal/l) | continuous | 757 | 0.168 | 0.131 | 0.20 | 0.57 |
| Aspartate aminotransferase (µkatal/l) | continuous | 757 | 0.336 | 0.183 | 0.07 | 0.57 |
| Serum amylase (alpha-amylase) (μkatal/l) | continuous | 757 | -0.147 | 0.091 | 0.11 | 0.57 |
| Gamma-glutamyl transferase (µkatal/l) | continuous | 757 | 0.061 | 0.031 | 0.049 | 0.57 |
| Lipase (μkatal/l) | continuous | 757 | -0.010 | 0.031 | 0.76 | 0.92 |
| Thyroid-stimulating hormone (mU/l) | continuous | 756 | 0.029 | 0.054 | 0.58 | 0.82 |
| Folic acid (ng/ml) | continuous | 755 | 0.004 | 0.006 | 0.49 | 0.82 |
| Anti-thyroid peroxidase antibody (U/ml) | continuous | 752 | 0.0002 | 0.0002 | 0.33 | 0.68 |
| ***Somatometric variables*** |  |  |  |  |  |  |
| Height (cm) | continuous | 756 | -0.006 | 0.006 | 0.28 | 0.61 |
| Weight (kg) | continuous | 756 | 0.002 | 0.003 | 0.47 | 0.82 |
| Waist circumference (cm) | continuous | 756 | 0.005 | 0.003 | 0.10 | 0.57 |
| Hip circumference (cm) | continuous | 756 | -0.002 | 0.004 | 0.53 | 0.82 |

B, beta-coefficients from linear regression models; N, sample size; SE, standard error. * adjusted for multiple testing according to the Benjamini-Hochberg method (for multicategorical exposures, p values from the global test were considered)

**Supplementary Table 3. Linear regression model results evaluating associations of various variables with saliva levels of X-23662.**

| Variable | Level | N | B | SE | P value | q value* |
| --- | --- | --- | --- | --- | --- | --- |
| ***Medical diseases*** |  |  |  |  |  |  |
| **Smoking (ref. never smoker)** | Ex-smoker | 926 | 0.007 | 0.074 | 0.92 |  |
|  | **Current smoker** |  | **0.282** | **0.093** | **0.002** | **0.031** |
| **Known or diagnosed diabetes mellitus** | **no/yes** | **892** | **0.349** | **0.122** | **0.004** | **0.030** |
| **Known diabetes mellitus** | **no/yes** | **889** | **0.416** | **0.135** | **0.002** | **0.023** |
| Self-reported general state of health (Excellent or very good, ref.) | good | 927 | -0.086 | 0.079 | 0.28 |  |
|  | less good or bad |  | 0.014 | 0.124 | 0.91 | 0.64 |
| **Has a raised or high blood pressure (hypertension) been determined medically since the last SHIP-study?** | **no/yes** | **925** | **0.207** | **0.070** | **0.003** | **0.026** |
| Heart rate (bpm) | continuous | 924 | 0.003 | 0.003 | 0.38 | 0.60 |
| Diastolic blood pressure (mmHg) | continuous | 922 | 0.007 | 0.003 | 0.03 | 0.13 |
| **Systolic blood pressure (mmHg)** | **continuous** | **920** | **0.006** | **0.002** | **0.003** | **0.026** |
| Do you often suffer from a bad taste in your mouth? | no/yes | 926 | 0.051 | 0.093 | 0.58 | 0.72 |
| Do you often suffer from a bad breath? | no/yes | 868 | 0.024 | 0.098 | 0.80 | 0.85 |
| Did you suffer more than once from pancreatitis? | no/yes | 921 | -0.832 | 0.375 | 0.03 | 0.11 |
| Have you had one of the following medical problems (diseases) within the last 12 months? |  |  |  |  |  |  |
| Varicose veins, thrombosis, phlebitis | no/yes | 925 | 0.001 | 0.122 | 0.99 | 0.99 |
| Inflammatory skin diseases (e.g. acne) | no/yes | 922 | -0.084 | 0.115 | 0.47 | 0.65 |
| degeneration of the joints, e.g. arthrosis of the hip, knee, shoulder, or foot joints | no/yes | 921 | -0.105 | 0.081 | 0.20 | 0.45 |
| inflammatory joint diseases, e.g. chronic polyarthritis | no/yes | 916 | 0.066 | 0.176 | 0.71 | 0.80 |
| osteoporosis, i.e. reduced bone density | no/yes | 917 | 0.052 | 0.194 | 0.79 | 0.85 |
| gastritis | no/yes | 922 | -0.183 | 0.176 | 0.30 | 0.52 |
| hepatitis | no/yes | 926 | 0.312 | 0.574 | 0.59 | 0.72 |
| fatty liver | no/yes | 923 | 0.009 | 0.121 | 0.94 | 0.95 |
| increased level of blood lipids (cholesterol, triglycerides) | no/yes | 918 | 0.055 | 0.083 | 0.50 | 0.65 |
| gout or increased uric acid | no/yes | 919 | 0.288 | 0.130 | 0.03 | 0.11 |
| chronic bronchitis, i.e. coughing phlegm in the morning on most days, at least 3 months within the last 2 years? | no/yes | 925 | -189 | 0.171 | 0.27 | 0.52 |
| Have you ever had cancer? | no/yes | 924 | -0.011 | 0.150 | 0.94 | 0.95 |
| Do you suffer from a medically determined chronic lung disease? | no/yes | 926 | 0.0660 | 0.173 | 0.70 | 0.80 |
| Do you suffer from psoriasis? | no/yes | 924 | 0.153 | 0.160 | 0.34 | 0.56 |
| Have you suffered from gastritis within the last six months? | no/yes | 921 | 0.051 | 0.184 | 0.78 | 0.85 |
| Have you ever had a stomach (gastric) ulcer? | no/yes | 924 | -0.103 | 0.120 | 0.39 | 0.60 |
| ***Laboratory variables*** |  |  |  |  |  |  |
| Sodium (mmol/l) | continuous | 927 | 0.021 | 0.014 | 0.15 | 0.41 |
| Potassium (mmol/l) | continuous | 927 | 0.130 | 0.090 | 0.15 | 0.41 |
| Serum creatinine (µmol/l) | continuous | 927 | 0.003 | 0.002 | 0.18 | 0.44 |
| Serum uric acid (µmol/l) | continuous | 927 | 0.001 | 0.001 | 0.07 | 0.24 |
| Serum glucose (mmol/l) | continuous | 927 | 0.045 | 0.024 | 0.06 | 0.19 |
| Cystatin C (mg/l) | continuous | 926 | 0.286 | 0.274 | 0.30 | 0.52 |
| Serum calcium, total (mmol/l) | continuous | 927 | 0.583 | 0.350 | 0.10 | 0.30 |
| Magnesium (mmol/l) | continuous | 927 | -0.819 | 0.356 | 0.02 | 0.11 |
| QUICK/Thromboplastin time (%) | continuous | 900 | -0.004 | 0.002 | 0.047 | 0.17 |
| Thromboplastin time international normalized ratio | continuous | 921 | 0.334 | 0.137 | 0.02 | 0.09 |
| Partial thromboplastin time (s) | continuous | 923 | 0.016 | 0.011 | 0.16 | 0.42 |
| Fibrinogen acc. to Clauss (g/l) | continuous | 922 | 0.051 | 0.048 | 0.29 | 0.52 |
| Haemoglobin A1c (%) | continuous | 927 | 0.094 | 0.045 | 0.04 | 0.15 |
| Creatinine kinase, total (µkatal/l) | continuous | 927 | 0.031 | 0.023 | 0.19 | 0.45 |
| Lactate dehydrogenase (µkatal/l) | continuous | 927 | 0.096 | 0.076 | 0.21 | 0.46 |
| **Leukocytes (Gpt/l)** | **continuous** | **926** | **0.087** | **0.019** | **<0.001** | **0.0003** |
| Erythrocytes (tpt/l) | continuous | 926 | 0.100 | 0.092 | 0.28 | 0.52 |
| Haemoglobin (mmol/l) | continuous | 926 | 0.021 | 0.052 | 0.68 | 0.80 |
| Haematocrit | continuous | 926 | 1.964 | 1.149 | 0.09 | 0.28 |
| Mean corpuscular volume (fl) | continuous | 926 | 0.006 | 0.008 | 0.44 | 0.64 |
| Mean corpuscular haemoglobin (fmol) | continuous | 926 | -0.332 | 0.343 | 0.33 | 0.56 |
| **Mean corpuscular haemoglobin concentration (mmol/l)** | **continuous** | **926** | **-0.177** | **0.057** | **0.002** | **0.023** |
| Red cell distribution width (%) | continuous | 926 | 0.044 | 0.057 | 0.26 | 0.52 |
| Thrombocytes (gpt/l) | continuous | 926 | 0.001 | 0.001 | 0.02 | 0.11 |
| Mean platelet volume (fl) | continuous | 926 | 0.038 | 0.028 | 0.17 | 0.43 |
| Triglycerides (total) (mmol/l) | continuous | 927 | 0.030 | 0.027 | 0.26 | 0.52 |
| Cholesterol (total) (mmol/l) | continuous | 925 | .022 | 0.031 | 0.48 | 0.65 |
| LDL-cholesterol (mmol/l) | continuous | 926 | 0.019 | 0.036 | 0.60 | 0.72 |
| HDL-cholesterol (mmol/l) | continuous | 926 | -0.084 | 0.096 | 0.38 | 0.60 |
| Alanine aminotransferase (µkatal/l) | continuous | 927 | 0.089 | 0.124 | 0.48 | 0.65 |
| Aspartate aminotransferase (µkatal/l) | continuous | 925 | 0.115 | 0.174 | 0.51 | 0.65 |
| Serum amylase (alpha-amylase) (μkatal/l) | continuous | 927 | -0.093 | 0.081 | 0.26 | 0.52 |
| Gamma-glutamyl transferase (µkatal/l) | continuous | 927 | 0.019 | 0.030 | 0.53 | 0.67 |
| Lipase (μkatal/l) | continuous | 927 | -0.010 | 0.028 | 0.72 | 0.80 |
| Thyroid-stimulating hormone (mU/l) | continuous | 926 | 0.009 | 0.042 | 0.82 | 0.86 |
| Folic acid (ng/ml) | continuous | 924 | -0.004 | 0.005 | 0.49 | 0.65 |
| Anti-thyroid peroxidase antibody (U/ml) | continuous | 920 | 0.001 | 0.0002 | 0.01 | 0.07 |
| ***Somatometric variables*** |  |  |  |  |  |  |
| Height (cm) | continuous | 926 | 0.004 | 0.005 | 0.40 | 0.60 |
| **Weight (kg)** | **continuous** | **926** | **0.009** | **0.002** | **0.0001** | **0.003** |
| **Waist circumference (cm)** | **continuous** | **926** | **0.013** | **0.003** | **<0.001** | **0.0003** |
| **Hip circumference (cm)** | **continuous** | **926** | **0.012** | **0.003** | **0.0004** | **0.007** |

B, beta-coefficients from linear regression models; N, sample size; SE, standard error. * adjusted for multiple testing according to the Benjamini-Hochberg method (for multicategorical exposures, p values from the global test were considered)

**Supplementary Table 4. Linear regression model results evaluating associations of various variables with saliva levels of butyrylputrescine.**

| Variable | Level | N | B | SE | P value | q value* |
| --- | --- | --- | --- | --- | --- | --- |
| ***Medical diseases*** |  |  |  |  |  |  |
| **Smoking (ref. never smoker)** | **Ex-smoker** | **752** | **-0.126** | **0.083** | **0.13** |  |
|  | **Current smoker** |  | **0.375** | **0.098** | **<0.001** | **0.00013** |
| Known or diagnosed diabetes mellitus | no/yes | 722 | 0.200 | 0.132 | 0.13 | 0.52 |
| Known diabetes mellitus | no/yes | 719 | 0.183 | 0.146 | 0.21 | 0.57 |
| Self-reported general state of health (Excellent or very good, ref.) | good | 752 | -0.062 | 0.088 | 0.48 |  |
|  | less good or bad |  | -0.181 | 0.137 | 0.19 | 0.72 |
| Has a raised or high blood pressure (hypertension) been determined medically since the last SHIP-study? | no/yes | 751 | 0.046 | 0.079 | 0.56 | 0.79 |
| Heart rate (bpm) | continuous | 749 | 0.007 | 0.003 | 0.05 | 0.25 |
| Diastolic blood pressure (mmHg) | continuous | 748 | -0.003 | 0.004 | 0.39 | 0.72 |
| Systolic blood pressure (mmHg) | continuous | 746 | 0.001 | 0.002 | 0.52 | 0.79 |
| Do you often suffer from a bad taste in your mouth? | no/yes | 752 | 0.212 | 0.102 | 0.039 | 0.25 |
| **Do you often suffer from a bad breath?** | **no/yes** | **705** | **0.324** | **0.107** | **0.002** | **0.033** |
| Did you suffer more than once from pancreatitis? | no/yes | 746 | -1.059 | 0.405 | 0.009 | 0.10 |
| Have you had one of the following medical problems (diseases) within the last 12 months? |  |  |  |  |  |  |
| Varicose veins, thrombosis, phlebitis | no/yes | 751 | -0.148 | 0.139 | 0.29 | 0.66 |
| Inflammatory skin diseases (e.g. acne) | no/yes | 748 | -0.066 | 0.135 | 0.63 | 0.79 |
| degeneration of the joints, e.g. arthrosis of the hip, knee, shoulder, or foot joints | no/yes | 750 | -0.182 | 0.090 | 0.04 | 0.25 |
| inflammatory joint diseases, e.g. chronic polyarthritis | no/yes | 743 | -0.205 | 0.203 | 0.31 | 0.66 |
| osteoporosis, i.e. reduced bone density | no/yes | 746 | -0.008 | 0.211 | 0.97 | 0.97 |
| gastritis | no/yes | 749 | -0.051 | 0.199 | 0.80 | 0.85 |
| hepatitis | no/yes | 752 | 0.728 | 0.576 | 0.21 | 0.57 |
| fatty liver | no/yes | 750 | -0.023 | 0.139 | 0.87 | 0.91 |
| increased level of blood lipids (cholesterol, triglycerides) | no/yes | 747 | 0.047 | 0.092 | 0.61 | 0.79 |
| gout or increased uric acid | no/yes | 747 | 0.036 | 0.143 | 0.80 | 0.85 |
| chronic bronchitis, i.e. coughing phlegm in the morning on most days, at least 3 months within the last 2 years? | no/yes | 751 | -0.204 | 0.202 | 0.61 | 0.79 |
| Have you ever had cancer? | no/yes | 750 | -0.146 | 0.170 | 0.39 | 0.72 |
| Do you suffer from a medically determined chronic lung disease? | no/yes | 752 | -0.014 | 0.192 | 0.94 | 0.95 |
| Do you suffer from psoriasis? | no/yes | 750 | 0.202 | 0.186 | 0.28 | 0.66 |
| Have you suffered from gastritis within the last six months? | no/yes | 748 | 0.074 | 0.202 | 0.72 | 0.83 |
| Have you ever had a stomach (gastric) ulcer? | no/yes | 750 | 0.069 | 0.136 | 0.61 | 0.79 |
| ***Laboratory variables*** |  |  |  |  |  |  |
| **Sodium (mmol/l)** | **continuous** | **752** | **0.051** | **0.016** | **0.002** | **0.029** |
| Potassium (mmol/l) | continuous | 752 | 0.225 | 0.098 | 0.02 | 0.19 |
| Serum creatinine (µmol/l) | continuous | 752 | -0.004 | 0.002 | 0.11 | 0.47 |
| Serum uric acid (µmol/l) | continuous | 752 | -0.001 | 0.001 | 0.37 | 0.72 |
| Serum glucose (mmol/l) | continuous | 752 | 0.038 | 0.028 | 0.18 | 0.57 |
| Cystatin C (mg/l) | continuous | 751 | 0.027 | 0.302 | 0.93 | 0.95 |
| Serum calcium, total (mmol/l) | continuous | 752 | 0.290 | 0.374 | 0.44 | 0.75 |
| Magnesium (mmol/l) | continuous | 752 | 0.125 | 0.389 | 0.75 | 0.85 |
| QUICK/Thromboplastin time (%) | continuous | 730 | -0.002 | 0.002 | 0.38 | 0.72 |
| Thromboplastin time international normalized ratio | continuous | 746 | 0.132 | 0.141 | 0.35 | 0.72 |
| Partial thromboplastin time (s) | continuous | 748 | 0.006 | 0.012 | 0.64 | 0.79 |
| **Fibrinogen acc. to Clauss (g/l)** | **continuous** | **747** | **0.169** | **0.053** | **0.001** | **0.026** |
| Haemoglobin A1c (%) | continuous | 752 | 0.086 | 0.058 | 0.14 | 0.53 |
| Creatinine kinase, total (µkatal/l) | continuous | 752 | -0.031 | 0.024 | 0.21 | 0.57 |
| Lactate dehydrogenase (µkatal/l) | continuous | 752 | -0.121 | 0.085 | 0.16 | 0.57 |
| **Leukocytes (Gpt/l)** | **continuous** | **752** | **0.088** | **0.020** | **<0.001** | **0.0004** |
| Erythrocytes (tpt/l) | continuous | 752 | 0.040 | 0.103 | 0.70 | 0.82 |
| Haemoglobin (mmol/l) | continuous | 752 | 0.015 | 0.058 | 0.80 | 0.85 |
| Haematocrit | continuous | 752 | 1.315 | 1.284 | 0.31 | 0.66 |
| Mean corpuscular volume (fl) | continuous | 752 | 0.005 | 0.009 | 0.55 | 0.79 |
| Mean corpuscular haemoglobin (fmol) | continuous | 752 | -0.177 | 0.378 | 0.64 | 0.79 |
| Mean corpuscular haemoglobin concentration (mmol/l) | continuous | 752 | -0.128 | 0.064 | 0.04 | 0.25 |
| Red cell distribution width (%) | continuous | 752 | 0.088 | 0.043 | 0.04 | 0.25 |
| Thrombocytes (gpt/l) | continuous | 752 | 0.0004 | 0.001 | 0.60 | 0.79 |
| Mean platelet volume (fl) | continuous | 752 | 0.020 | 0.031 | 0.53 | 0.79 |
| Triglycerides (total) (mmol/l) | continuous | 752 | 0.013 | 0.029 | 0.65 | 0.79 |
| Cholesterol (total) (mmol/l) | continuo us | 750 | -0.036 | 0.034 | 0.28 | 0.66 |
| LDL-cholesterol (mmol/l) | continuous | 752 | -0.023 | 0.039 | 0.56 | 0.79 |
| HDL-cholesterol (mmol/l) | continuous | 752 | -0.203 | 0.104 | 0.05 | 0.25 |
| Alanine aminotransferase (µkatal/l) | continuous | 752 | 0.057 | 0.140 | 0.69 | 0.82 |
| Aspartate aminotransferase (µkatal/l) | continuous | 751 | 0.261 | 0.209 | 0.21 | 0.57 |
| Serum amylase (alpha-amylase) (μkatal/l) | continuous | 752 | -0.105 | 0.087 | 0.23 | 0.60 |
| Gamma-glutamyl transferase (µkatal/l) | continuous | 752 | 0.124 | 0.053 | 0.02 | 0.17 |
| Lipase (μkatal/l) | continuous | 752 | -0.014 | 0.032 | 0.65 | 0.79 |
| Thyroid-stimulating hormone (mU/l) | continuous | 751 | -0.038 | 0.047 | 0.41 | 0.72 |
| Folic acid (ng/ml) | continuous | 749 | -0.001 | 0.005 | 0.79 | 0.85 |
| Anti-thyroid peroxidase antibody (U/ml) | continuous | 747 | 0.0001 | 0.0002 | 0.61 | 0.79 |
| ***Somatometric variables*** |  |  |  |  |  |  |
| Height (cm) | continuous | 751 | -0.003 | 0.006 | 0.55 | 0.79 |
| Weight (kg) | continuous | 751 | 0.003 | 0.003 | 0.24 | 0.60 |
| Waist circumference (cm) | continuous | 751 | 0.006 | 0.003 | 0.08 | 0.34 |
| Hip circumference (cm) | continuous | 751 | 0.005 | 0.004 | 0.17 | 0.57 |

B, beta-coefficients from linear regression models; N, sample size; SE, standard error. * adjusted for multiple testing according to the Benjamini-Hochberg method (for multicategorical exposures, p values from the global test were considered)

**Supplementary Table 5. Linear regression model results evaluating associations of various variables with saliva levels of N,N-dimethyl-5-aminovalerate.**

| Variable | Level | N | B | SE | P value | q value* |
| --- | --- | --- | --- | --- | --- | --- |
| ***Medical diseases*** |  |  |  |  |  |  |
| **Smoking (ref. never smoker)** | **Ex-smoker** | **595** | **-0.010** | **0.096** | **0.91** |  |
|  | **Current smoker** |  | **0.455** | **0.110** | **<0.001** | **0.001** |
| Known or diagnosed diabetes mellitus | no/yes | 568 | 0.329 | 0.153 | 0.03 | 0.27 |
| Known diabetes mellitus | no/yes | 565 | 0.357 | 0.171 | 0.04 | 0.29 |
| Self-reported general state of health (Excellent or very good, ref.) | good | 595 | -0.169 | 0.099 | 0.09 |  |
|  | less good or bad |  | -0.423 | 0.154 | 0.006 | 0.20 |
| Has a raised or high blood pressure (hypertension) been determined medically since the last SHIP-study? | no/yes | 594 | 0.230 | 0.089 | 0.01 | 0.17 |
| Heart rate (bpm) | continuous | 593 | -0.002 | 0.004 | 0.68 | 0.93 |
| Diastolic blood pressure (mmHg) | continuous | 591 | -0.003 | 0.004 | 0.51 | 0.91 |
| Systolic blood pressure (mmHg) | continuous | 590 | 0.003 | 0.002 | 0.28 | 0.83 |
| Do you often suffer from a bad taste in your mouth? | no/yes | 595 | 0.291 | 0.112 | 0.01 | 0.17 |
| Do you often suffer from a bad breath? | no/yes | 555 | 0.281 | 0.121 | 0.02 | 0.20 |
| Did you suffer more than once from pancreatitis? | no/yes | 591 | -0.355 | 0.406 | 0.38 | 0.89 |
| Have you had one of the following medical problems (diseases) within the last 12 months? |  |  |  |  |  |  |
| Varicose veins, thrombosis, phlebitis | no/yes | 594 | 0.100 | 0.156 | 0.52 | 0.91 |
| Inflammatory skin diseases (e.g. acne) | no/yes | 591 | 0.068 | 0.150 | 0.65 | 0.93 |
| degeneration of the joints, e.g. arthrosis of the hip, knee, shoulder, or foot joints | no/yes | 592 | -0.168 | 0.103 | 0.10 | 0.52 |
| inflammatory joint diseases, e.g. chronic polyarthritis | no/yes | 586 | -0.166 | 0.228 | 0.47 | 0.91 |
| osteoporosis, i.e. reduced bone density | no/yes | 589 | 0.242 | 0.268 | 0.37 | 0.89 |
| gastritis | no/yes | 593 | -0.252 | 0.204 | 0.22 | 0.83 |
| hepatitis | no/yes | 595 | 0.513 | 0.581 | 0.38 | 0.89 |
| fatty liver | no/yes | 594 | 0.106 | 0.153 | 0.49 | 0.91 |
| increased level of blood lipids (cholesterol, triglycerides) | no/yes | 590 | -0.020 | 0.106 | 0.85 | 0.96 |
| gout or increased uric acid | no/yes | 590 | 0.037 | 0.166 | 0.83 | 0.96 |
| chronic bronchitis, i.e. coughing phlegm in the morning on most days, at least 3 months within the last 2 years? | no/yes | 595 | 0.027 | 0.228 | 0.91 | 0.97 |
| Have you ever had cancer? | no/yes | 594 | -0.361 | 0.177 | 0.04 | 0.29 |
| Do you suffer from a medically determined chronic lung disease? | no/yes | 595 | -0.237 | 0.204 | 0.25 | 0.83 |
| Do you suffer from psoriasis? | no/yes | 594 | 0.096 | 0.205 | 0.64 | 0.93 |
| Have you suffered from gastritis within the last six months? | no/yes | 592 | -0.069 | 0.207 | 0.74 | 0.93 |
| Have you ever had a stomach (gastric) ulcer? | no/yes | 593 | 0.063 | 0.160 | 0.70 | 0.93 |
| ***Laboratory variables*** |  |  |  |  |  |  |
| Sodium (mmol/l) | continuous | 595 | -0.012 | 0.018 | 0.51 | 0.91 |
| Potassium (mmol/l) | continuous | 595 | 0.217 | 0.112 | 0.05 | 0.33 |
| Serum creatinine (µmol/l) | continuous | 595 | -0.001 | 0.003 | 0.72 | 0.93 |
| Serum uric acid (µmol/l) | continuous | 595 | 0.0001 | 0.001 | 0.86 | 0.96 |
| Serum glucose (mmol/l) | continuous | 595 | 0.030 | 0.028 | 0.27 | 0.83 |
| Cystatin C (mg/l) | continuous | 594 | 0.224 | 0.354 | 0.53 | 0.91 |
| Serum calcium, total (mmol/l) | continuous | 595 | 0.149 | 0.426 | 0.73 | 0.93 |
| Magnesium (mmol/l) | continuous | 595 | 0.419 | 0.452 | 0.35 | 0.89 |
| QUICK/Thromboplastin time (%) | continuous | 577 | -0.0001 | 0.003 | 0.96 | 0.97 |
| Thromboplastin time international normalized ratio | continuous | 592 | 0.042 | 0.173 | 0.81 | 0.96 |
| Partial thromboplastin time (s) | continuous | 593 | 0.008 | 0.013 | 0.56 | 0.91 |
| Fibrinogen acc. to Clauss (g/l) | continuous | 592 | 0.076 | 0.059 | 0.20 | 0.83 |
| Haemoglobin A1c (%) | continuous | 595 | -0.019 | 0.054 | 0.72 | 0.93 |
| Creatinine kinase, total (µkatal/l) | continuous | 595 | 0.015 | 0.037 | 0.68 | 0.93 |
| Lactate dehydrogenase (µkatal/l) | continuous | 595 | 0.072 | 0.094 | 0.44 | 0.91 |
| Leukocytes (Gpt/l) | continuous | 595 | 0.066 | 0.022 | 0.003 | 0.11 |
| Erythrocytes (tpt/l) | continuous | 595 | -0.128 | 0.113 | 0.26 | 0.83 |
| Haemoglobin (mmol/l) | continuous | 595 | -0.034 | 0.064 | 0.59 | 0.93 |
| Haematocrit | continuous | 595 | -0.166 | 1.428 | 0.91 | 0.97 |
| Mean corpuscular volume (fl) | continuous | 595 | 0.014 | 0.010 | 0.15 | 0.72 |
| Mean corpuscular haemoglobin (fmol) | continuous | 595 | 0.244 | 0.403 | 0.55 | 0.91 |
| Mean corpuscular haemoglobin concentration (mmol/l) | continuous | 595 | -0.090 | 0.071 | 0.21 | 0.83 |
| Red cell distribution width (%) | continuous | 595 | 0.003 | 0.048 | 0.96 | 0.97 |
| Thrombocytes (gpt/l) | continuous | 595 | 0.0004 | 0.001 | 0.62 | 0.93 |
| Mean platelet volume (fl) | continuous | 595 | 0.015 | 0.035 | 0.67 | 0.93 |
| Triglycerides (total) (mmol/l) | continuous | 595 | 0.006 | 0.031 | 0.85 | 0.96 |
| Cholesterol (total) (mmol/l) | continuous | 594 | -0.036 | 0.039 | 0.35 | 0.89 |
| LDL-cholesterol (mmol/l) | continuous | 595 | -0.052 | 0.045 | 0.25 | 0.83 |
| HDL-cholesterol (mmol/l) | continuous | 595 | -0.032 | 0.119 | 0.79 | 0.96 |
| Alanine aminotransferase (µkatal/l) | continuous | 595 | -0.037 | 0.145 | 0.80 | 0.96 |
| Aspartate aminotransferase (µkatal/l) | continuous | 593 | -0.009 | 0.198 | 0.97 | 0.97 |
| Serum amylase (alpha-amylase) (μkatal/l) | continuous | 595 | -0.361 | 0.146 | 0.01 | 0.19 |
| Gamma-glutamyl transferase (µkatal/l) | continuous | 595 | 0.028 | 0.032 | 0.38 | 0.89 |
| Lipase (μkatal/l) | continuous | 595 | -0.002 | 0.035 | 0.96 | 0.97 |
| Thyroid-stimulating hormone (mU/l) | continuous | 594 | 0.044 | 0.061 | 0.47 | 0.91 |
| Folic acid (ng/ml) | continuous | 594 | -0.004 | 0.007 | 0.56 | 0.91 |
| Anti-thyroid peroxidase antibody (U/ml) | continuous | 593 | 0.0005 | 0.0002 | 0.06 | 0.35 |
| ***Somatometric variables*** |  |  |  |  |  |  |
| Height (cm) | continuous | 594 | -0.001 | 0.006 | 0.90 | 0.97 |
| Weight (kg) | continuous | 594 | 0.002 | 0.003 | 0.55 | 0.91 |
| Waist circumference (cm) | continuous | 594 | 0.005 | 0.004 | 0.16 | 0.73 |
| Hip circumference (cm) | continuous | 594 | 0.003 | 0.004 | 0.53 | 0.91 |

B, beta-coefficients from linear regression models; N, sample size; SE, standard error. * adjusted for multiple testing according to the Benjamini-Hochberg method (for multicategorical exposures, p values from the global test were considered)

**Supplementary Table 6. Linear regression model results evaluating associations of various variables with saliva levels of N-acetylcadaverine.**

| Variable | Level | N | B | SE | P value | q value* |
| --- | --- | --- | --- | --- | --- | --- |
| ***Medical diseases*** |  |  |  |  |  |  |
| **Smoking (ref. never smoker)** | **Ex-smoker** | **969** | **0.058** | **0.072** | **0.42** |  |
|  | **Current smoker** |  | **0.290** | **0.090** | **0.001** | **0.047** |
| Known or diagnosed diabetes mellitus | no/yes | 932 | 0.190 | 0.116 | 0.10 | 0.28 |
| Known diabetes mellitus | no/yes | 929 | 0.298 | 0.129 | 0.021 | 0.18 |
| Self-reported general state of health (Excellent or very good, ref.) | good | 970 | -0.078 | 0.077 | 0.31 |  |
|  | less good or bad |  | 0.066 | 0.121 | 0.59 | 0.56 |
| Has a raised or high blood pressure (hypertension) been determined medically since the last SHIP-study? | no/yes | 968 | 0.144 | 0.069 | 0.04 | 0.21 |
| Heart rate (bpm) | continuous | 967 | 0.005 | 0.003 | 0.11 | 0.30 |
| Diastolic blood pressure (mmHg) | continuous | 965 | 0.006 | 0.003 | 0.07 | 0.25 |
| **Systolic blood pressure (mmHg)** | **continuous** | **963** | **0.006** | **0.002** | **0.003** | **0.034** |
| Do you often suffer from a bad taste in your mouth? | no/yes | 970 | 0.033 | 0.091 | 0.71 | 0.81 |
| Do you often suffer from a bad breath? | no/yes | 911 | -0.020 | 0.094 | 0.83 | 0.88 |
| Did you suffer more than once from pancreatitis? | no/yes | 963 | -0.486 | 0.372 | 0.19 | 0.38 |
| Have you had one of the following medical problems (diseases) within the last 12 months? |  |  |  |  |  |  |
| Varicose veins, thrombosis, phlebitis | no/yes | 968 | -0.046 | 0.121 | 0.71 | 0.81 |
| Inflammatory skin diseases (e.g. acne) | no/yes | 965 | -0.033 | 0.113 | 0.77 | 0.85 |
| degeneration of the joints, e.g. arthrosis of the hip, knee, shoulder, or foot joints | no/yes | 964 | -0.146 | 0.079 | 0.06 | 0.25 |
| inflammatory joint diseases, e.g. chronic polyarthritis | no/yes | 959 | 0.075 | 0.173 | 0.67 | 0.80 |
| osteoporosis, i.e. reduced bone density | no/yes | 960 | 0.032 | 0.190 | 0.86 | 0.89 |
| gastritis | no/yes | 965 | -0.147 | 0.175 | 0.40 | 0.65 |
| hepatitis | no/yes | 969 | 0.399 | 0.572 | 0.49 | 0.69 |
| fatty liver | no/yes | 966 | 0.132 | 0.120 | 0.27 | 0.50 |
| increased level of blood lipids (cholesterol, triglycerides) | no/yes | 961 | 0.046 | 0.081 | 0.57 | 0.72 |
| gout or increased uric acid | no/yes | 962 | 0.148 | 0.128 | 0.25 | 0.48 |
| chronic bronchitis, i.e. coughing phlegm in the morning on most days, at least 3 months within the last 2 years? | no/yes | 968 | -0.106 | 0.168 | 0.53 | 0.72 |
| Have you ever had cancer? | no/yes | 967 | 0.049 | 0.146 | 0.74 | 0.83 |
| Do you suffer from a medically determined chronic lung disease? | no/yes | 969 | 0.091 | 0.170 | 0.59 | 0.73 |
| Do you suffer from psoriasis? | no/yes | 967 | 0.024 | 0.158 | 0.88 | 0.89 |
| Have you suffered from gastritis within the last six months? | no/yes | 964 | 0.144 | 0.183 | 0.43 | 0.65 |
| Have you ever had a stomach (gastric) ulcer? | no/yes | 967 | -0.073 | 0.119 | 0.54 | 0.72 |
| ***Laboratory variables*** |  |  |  |  |  |  |
| Sodium (mmol/l) | continuous | 970 | 0.020 | 0.014 | 0.15 | 0.35 |
| Potassium (mmol/l) | continuous | 970 | 0.052 | 0.087 | 0.55 | 0.72 |
| Serum creatinine (µmol/l) | continuous | 970 | 0.001 | 0.002 | 0.55 | 0.72 |
| Serum uric acid (µmol/l) | continuous | 970 | 0.001 | 0.0005 | 0.10 | 0.28 |
| Serum glucose (mmol/l) | continuous | 970 | 0.031 | 0.023 | 0.17 | 0.35 |
| Cystatin C (mg/l) | continuous | 969 | 0.472 | 0.269 | 0.08 | 0.25 |
| Serum calcium, total (mmol/l) | continuous | 970 | 0.315 | 0.334 | 0.35 | 0.61 |
| Magnesium (mmol/l) | continuous | 970 | -0.605 | 0.348 | 0.08 | 0.25 |
| QUICK/Thromboplastin time (%) | continuous | 942 | -0.004 | 0.002 | 0.08 | 0.25 |
| Thromboplastin time international normalized ratio | continuous | 964 | 0.283 | 0.137 | 0.04 | 0.21 |
| Partial thromboplastin time (s) | continuous | 966 | 0.016 | 0.011 | 0.14 | 0.35 |
| Fibrinogen acc. to Clauss (g/l) | continuous | 965 | 0.088 | 0.046 | 0.06 | 0.25 |
| Haemoglobin A1c (%) | continuous | 970 | 0.067 | 0.044 | 0.13 | 0.34 |
| Creatinine kinase, total (µkatal/l) | continuous | 970 | 0.016 | 0.023 | 0.49 | 0.69 |
| Lactate dehydrogenase (µkatal/l) | continuous | 970 | 0.129 | 0.074 | 0.08 | 0.25 |
| **Leukocytes (Gpt/l)** | **continuous** | **969** | **0.099** | **0.018** | **<0.001** | **<0.001** |
| Erythrocytes (tpt/l) | continuous | 969 | 0.162 | 0.090 | 0.07 | 0.25 |
| Haemoglobin (mmol/l) | continuous | 969 | 0.042 | 0.051 | 0.41 | 0.65 |
| Haematocrit | continuous | 969 | 2.501 | 1.125 | 0.03 | 0.18 |
| Mean corpuscular volume (fl) | continuous | 969 | 0.003 | 0.008 | 0.70 | 0.81 |
| Mean corpuscular haemoglobin (fmol) | continuous | 969 | -0.481 | 0.338 | 0.16 | 0.35 |
| **Mean corpuscular haemoglobin concentration (mmol/l)** | **continuous** | **969** | **-0.184** | **0.056** | **0.001** | **0.015** |
| Red cell distribution width (%) | continuous | 969 | 0.080 | 0.039 | 0.04 | 0.21 |
| Thrombocytes (gpt/l) | continuous | 969 | 0.001 | 0.001 | 0.06 | 0.25 |
| Mean platelet volume (fl) | continuous | 969 | 0.037 | 0.027 | 0.17 | 0.35 |
| Triglycerides (total) (mmol/l) | continuous | 970 | 0.023 | 0.026 | 0.38 | 0.65 |
| Cholesterol (total) (mmol/l) | continuous | 968 | 0.005 | 0.030 | 0.86 | 0.89 |
| LDL-cholesterol (mmol/l) | continuous | 969 | 0.004 | 0.035 | 0.91 | 0.91 |
| HDL-cholesterol (mmol/l) | continuous | 969 | -0.129 | 0.092 | 0.16 | 0.35 |
| Alanine aminotransferase (µkatal/l) | continuous | 970 | 0.095 | 0.121 | 0.43 | 0.65 |
| Aspartate aminotransferase (µkatal/l) | continuous | 968 | 0.044 | 0.172 | 0.80 | 0.86 |
| Serum amylase (alpha-amylase) (μkatal/l) | continuous | 970 | -0.181 | 0.080 | 0.02 | 0.18 |
| Gamma-glutamyl transferase (µkatal/l) | continuous | 970 | 0.018 | 0.030 | 0.56 | 0.72 |
| Lipase (μkatal/l) | continuous | 970 | -0.019 | 0.027 | 0.48 | 0.69 |
| Thyroid-stimulating hormone (mU/l) | continuous | 969 | -0.048 | 0.040 | 0.23 | 0.45 |
| Folic acid (ng/ml) | continuous | 967 | -0.002 | 0.005 | 0.62 | 0.75 |
| Anti-thyroid peroxidase antibody (U/ml) | continuous | 963 | 0.0002 | 0.0002 | 0.43 | 0.65 |
| ***Somatometric variables*** |  |  |  |  |  |  |
| Height (cm) | continuous | 969 | 0.007 | 0.005 | 0.15 | 0.35 |
| **Weight (kg)** | **continuous** | **969** | **0.009** | **0.002** | **<0.001** | **0.002** |
| **Waist circumference (cm)** | **continuous** | **969** | **0.012** | **0.003** | **<0.001** | **<0.001** |
| **Hip circumference (cm)** | **continuous** | **969** | **0.012** | **0.003** | **<0.001** | **0.005** |

B, beta-coefficients from linear regression models; N, sample size; SE, standard error. * adjusted for multiple testing according to the Benjamini-Hochberg method (for multicategorical exposures, p values from the global test were considered)

**Supplementary Table 7. Linear regression model results evaluating associations of various variables with saliva levels of N6,N6,N6-trimethyllysine.**

| Variable | Level | N | B | SE | P value | q value* |
| --- | --- | --- | --- | --- | --- | --- |
| ***Medical diseases*** |  |  |  |  |  |  |
| Smoking (ref. never smoker) | Ex-smoker | 964 | 0.002 | 0.073 | 0.98 |  |
|  | Current smoker |  | -0.065 | 0.091 | 0.47 | 0.80 |
| Known or diagnosed diabetes mellitus | no/yes | 929 | 0.037 | 0.119 | 0.75 | 0.82 |
| Known diabetes mellitus | no/yes | 926 | 0.070 | 0.132 | 0.60 | 0.78 |
| Self-reported general state of health (Excellent or very good, ref.) | good | 965 | -0.152 | 0.077 | 0.049 |  |
|  | less good or bad |  | -0.011 | 0.122 | 0.93 | 0.31 |
| Has a raised or high blood pressure (hypertension) been determined medically since the last SHIP-study? | no/yes | 963 | 0.173 | 0.069 | 0.01 | 0.10 |
| Heart rate (bpm) | continuous | 962 | 0.002 | 0.003 | 0.62 | 0.78 |
| Diastolic blood pressure (mmHg) | continuous | 960 | 0.007 | 0.003 | 0.02 | 0.13 |
| **Systolic blood pressure (mmHg)** | **continuous** | **958** | **0.006** | **0.002** | **0.002** | **0.023** |
| Do you often suffer from a bad taste in your mouth? | no/yes | 964 | 0.090 | 0.091 | 0.33 | 0.62 |
| Do you often suffer from a bad breath? | no/yes | 905 | 0.072 | 0.095 | 0.45 | 0.71 |
| Did you suffer more than once from pancreatitis? | no/yes | 958 | -0.208 | 0.377 | 0.58 | 0.78 |
| Have you had one of the following medical problems (diseases) within the last 12 months? |  |  |  |  |  |  |
| Varicose veins, thrombosis, phlebitis | no/yes | 963 | 0.230 | 0.124 | 0.06 | 0.23 |
| Inflammatory skin diseases (e.g. acne) | no/yes | 960 | -0.187 | 0.113 | 0.10 | 0.31 |
| degeneration of the joints, e.g. arthrosis of the hip, knee, shoulder, or foot joints | no/yes | 959 | -0.196 | 0.079 | 0.01 | 0.10 |
| inflammatory joint diseases, e.g. chronic polyarthritis | no/yes | 954 | -0.183 | 0.174 | 0.29 | 0.60 |
| osteoporosis, i.e. reduced bone density | no/yes | 955 | -0.054 | 0.191 | 0.78 | 0.82 |
| gastritis | no/yes | 960 | 0.126 | 0.179 | 0.48 | 0.73 |
| hepatitis | no/yes | 964 | 0.655 | 0.574 | 0.25 | 0.55 |
| fatty liver | no/yes | 961 | 0.171 | 0.121 | 0.16 | 0.42 |
| increased level of blood lipids (cholesterol, triglycerides) | no/yes | 956 | 0.073 | 0.081 | 0.37 | 0.65 |
| gout or increased uric acid | no/yes | 957 | 0.152 | 0.129 | 0.24 | 0.54 |
| chronic bronchitis, i.e. coughing phlegm in the morning on most days, at least 3 months within the last 2 years? | no/yes | 963 | 0.052 | 0.169 | 0.76 | 0.82 |
| Have you ever had cancer? | no/yes | 962 | -0.135 | 0.148 | 0.36 | 0.64 |
| Do you suffer from a medically determined chronic lung disease? | no/yes | 964 | 0.022 | 0.171 | 0.90 | 0.90 |
| Do you suffer from psoriasis? | no/yes | 962 | -0.041 | 0.158 | 0.80 | 0.82 |
| Have you suffered from gastritis within the last six months? | no/yes | 959 | 0.182 | 0.187 | 0.33 | 0.62 |
| Have you ever had a stomach (gastric) ulcer? | no/yes | 962 | -0.044 | 0.119 | 0.71 | 0.80 |
| ***Laboratory variables*** |  |  |  |  |  |  |
| Sodium (mmol/l) | continuous | 965 | 0.026 | 0.014 | 0.06 | 0.23 |
| Potassium (mmol/l) | continuous | 965 | 0.090 | 0.087 | 0.30 | 0.60 |
| Serum creatinine (µmol/l) | continuous | 965 | 0.001 | 0.002 | 0.67 | 0.80 |
| Serum uric acid (µmol/l) | continuous | 965 | 0.001 | 0.001 | 0.22 | 0.52 |
| Serum glucose (mmol/l) | continuous | 965 | -0.010 | 0.023 | 0.68 | 0.80 |
| Cystatin C (mg/l) | continuous | 964 | 0.364 | 0.270 | 0.18 | 0.44 |
| Serum calcium, total (mmol/l) | continuous | 965 | -0.131 | 0.339 | 0.70 | 0.80 |
| Magnesium (mmol/l) | continuous | 965 | -0.176 | 0.351 | 0.62 | 0.78 |
| QUICK/Thromboplastin time (%) | continuous | 937 | -0.003 | 0.002 | 0.15 | 0.51 |
| Thromboplastin time international normalized ratio | continuous | 959 | 0.273 | 0.137 | 0.047 | 0.21 |
| Partial thromboplastin time (s) | continuous | 961 | 0.021 | 0.011 | 0.05 | 0.23 |
| **Fibrinogen acc. to Clauss (g/l)** | **continuous** | **960** | **0.143** | **0.047** | **0.002** | **0.023** |
| Haemoglobin A1c (%) | continuous | 965 | 0.024 | 0.044 | 0.59 | 0.78 |
| Creatinine kinase, total (µkatal/l) | continuous | 965 | 0.014 | 0.023 | 0.54 | 0.78 |
| Lactate dehydrogenase (µkatal/l) | continuous | 965 | 0.118 | 0.074 | 0.11 | 0.33 |
| **Leukocytes (Gpt/l)** | **continuous** | **964** | **0.063** | **0.018** | **<0.001** | **0.021** |
| Erythrocytes (tpt/l) | continuous | 964 | 0.171 | 0.091 | 0.059 | 0.23 |
| Haemoglobin (mmol/l) | continuous | 964 | 0.027 | 0.051 | 0.60 | 0.78 |
| Haematocrit | continuous | 964 | 1.760 | 1.138 | 0.12 | 0.34 |
| Mean corpuscular volume (fl) | continuous | 964 | -0.008 | 0.008 | 0.29 | 0.60 |
| Mean corpuscular haemoglobin (fmol) | continuous | 964 | -0.785 | 0.339 | 0.02 | 0.13 |
| Mean corpuscular haemoglobin concentration (mmol/l) | continuous | 964 | -0.140 | 0.057 | 0.01 | 0.10 |
| Red cell distribution width (%) | continuous | 964 | 0.084 | 0.039 | 0.03 | 0.16 |
| Thrombocytes (gpt/l) | continuous | 964 | 0.001 | 0.001 | 0.36 | 0.64 |
| Mean platelet volume (fl) | continuous | 964 | 0.043 | 0.027 | 0.11 | 0.33 |
| Triglycerides (total) (mmol/l) | continuous | 965 | -0.019 | 0.026 | 0.46 | 0.71 |
| Cholesterol (total) (mmol/l) | continuous | 963 | -0.007 | 0.030 | 0.81 | 0.82 |
| LDL-cholesterol (mmol/l) | continuous | 964 | 0.018 | 0.035 | 0.61 | 0.78 |
| HDL-cholesterol (mmol/l) | continuous | 964 | -0.200 | 0.092 | 0.03 | 0.17 |
| Alanine aminotransferase (µkatal/l) | continuous | 965 | 0.163 | 0.121 | 0.18 | 0.44 |
| Aspartate aminotransferase (µkatal/l) | continuous | 963 | 0.109 | 0.173 | 0.53 | 0.78 |
| **Serum amylase (alpha-amylase) (μkatal/l)** | **continuous** | **965** | **-0.264** | **0.080** | **0.001** | **0.023** |
| Gamma-glutamyl transferase (µkatal/l) | continuous | 965 | 0.013 | 0.30 | 0.68 | 0.80 |
| Lipase (μkatal/l) | continuous | 965 | -0.013 | 0.027 | 0.63 | 0.78 |
| Thyroid-stimulating hormone (mU/l) | continuous | 964 | -0.033 | 0.40 | 0.42 | 0.70 |
| Folic acid (ng/ml) | continuous | 962 | -0.001 | 0.005 | 0.80 | 0.82 |
| Anti-thyroid peroxidase antibody (U/ml) | continuous | 958 | -0.0002 | 0.0002 | 0.38 | 0.65 |
| ***Somatometric variables*** |  |  |  |  |  |  |
| Height (cm) | continuous | 964 | 0.004 | 0.005 | 0.43 | 0.70 |
| **Weight (kg)** | **continuous** | **964** | **0.008** | **0.002** | **<0.001** | **0.015** |
| **Waist circumference (cm)** | **continuous** | **964** | **0.009** | **0.003** | **0.002** | **0.023** |
| **Hip circumference (cm)** | **continuous** | **964** | **0.010** | **0.003** | **0.003** | **0.032** |

B, beta-coefficients from linear regression models; N, sample size; SE, standard error. * adjusted for multiple testing according to the Benjamini-Hochberg method (for multicategorical exposures, p values from the global test were considered)

**Supplementary Table 8. Linear regression model results evaluating associations of various variables with saliva levels of N-acetyltaurine.**

| Variable | Level | N | B | SE | P value | q value* |
| --- | --- | --- | --- | --- | --- | --- |
| ***Medical diseases*** |  |  |  |  |  |  |
| **Smoking (ref. never smoker)** | **Ex-smoker** |  | **-0.163** | **0.072** | **0.03** |  |
|  | **Current smoker** |  | **-0.363** | **0.091** | **<0.001** | **0.02** |
| Known or diagnosed diabetes mellitus | no/yes | 917 | 0.087 | 0.119 | 0.46 | 0.64 |
| Known diabetes mellitus | no/yes | 914 | 0.159 | 0.131 | 0.23 | 0.59 |
| Self-reported general state of health (Excellent or very good, ref.) | good | 952 | -0.172 | 0.078 | 0.027 |  |
|  | less good or bad |  | -0.086 | 0.123 | 0.48 | 0.42 |
| Has a raised or high blood pressure (hypertension) been determined medically since the last SHIP-study? | no/yes | 951 | -0.017 | 0.070 | 0.80 | 0.86 |
| Heart rate (bpm) | continuous | 949 | -0.003 | 0.003 | 0.41 | 0.64 |
| Diastolic blood pressure (mmHg) | continuous | 948 | -0.003 | 0.003 | 0.43 | 0.64 |
| Systolic blood pressure (mmHg) | continuous | 946 | 0.002 | 0.002 | 0.44 | 0.64 |
| Do you often suffer from a bad taste in your mouth? | no/yes | 951 | -0.081 | 0.093 | 0.38 | 0.64 |
| Do you often suffer from a bad breath? | no/yes | 893 | -0.142 | 0.096 | 0.14 | 0.54 |
| Did you suffer more than once from pancreatitis? | no/yes | 946 | -0.464 | 0.374 | 0.22 | 0.59 |
| Have you had one of the following medical problems (diseases) within the last 12 months? |  |  |  |  |  |  |
| Varicose veins, thrombosis, phlebitis | no/yes | 951 | 0.044 | 0.122 | 0.72 | 0.86 |
| Inflammatory skin diseases (e.g. acne) | no/yes | 948 | 0.032 | 0.115 | 0.78 | 0.86 |
| degeneration of the joints, e.g. arthrosis of the hip, knee, shoulder, or foot joints | no/yes | 948 | 0.034 | 0.080 | 0.67 | 0.84 |
| inflammatory joint diseases, e.g. chronic polyarthritis | no/yes | 942 | -0.205 | 0.180 | 0.26 | 0.59 |
| osteoporosis, i.e. reduced bone density | no/yes | 943 | -0.040 | 0.191 | 0.83 | 0.88 |
| gastritis | no/yes | 948 | 0.370 | 0.187 | 0.048 | 0.33 |
| hepatitis | no/yes | 952 | 0.065 | 0.574 | 0.91 | 0.92 |
| fatty liver | no/yes | 949 | 0.060 | 0.123 | 0.62 | 0.81 |
| increased level of blood lipids (cholesterol, triglycerides) | no/yes | 944 | -0.061 | 0.082 | 0.46 | 0.64 |
| gout or increased uric acid | no/yes | 945 | 0.034 | 0.130 | 0.79 | 0.86 |
| chronic bronchitis, i.e. coughing phlegm in the morning on most days, at least 3 months within the last 2 years? | no/yes | 951 | -0.131 | 0.171 | 0.44 | 0.64 |
| Have you ever had cancer? | no/yes | 950 | 0.028 | 0.148 | 0.85 | 0.89 |
| Do you suffer from a medically determined chronic lung disease? | no/yes | 952 | 0.018 | 0.176 | 0.92 | 0.92 |
| Do you suffer from psoriasis? | no/yes | 950 | 0.113 | 0.156 | 0.47 | 0.64 |
| Have you suffered from gastritis within the last six months? | no/yes | 947 | 0.149 | 0.197 | 0.45 | 0.64 |
| Have you ever had a stomach (gastric) ulcer? | no/yes | 950 | 0.033 | 0.120 | 0.78 | 0.86 |
| ***Laboratory variables*** |  |  |  |  |  |  |
| Sodium (mmol/l) | continuous | 952 | 0.019 | 0.014 | 0.18 | 0.56 |
| Potassium (mmol/l) | continuous | 952 | 0.081 | 0.089 | 0.36 | 0.64 |
| Serum creatinine (µmol/l) | continuous | 952 | -0.002 | 0.002 | 0.35 | 0.64 |
| Serum uric acid (µmol/l) | continuous | 952 | -0.001 | 0.001 | 0.01 | 0.14 |
| Serum glucose (mmol/l) | continuous | 952 | -0.027 | 0.023 | 0.25 | 0.59 |
| Cystatin C (mg/l) | continuous | 951 | -0.188 | 0.271 | 0.49 | 0.65 |
| Serum calcium, total (mmol/l) | continuous | 952 | -0.147 | 0.338 | 0.66 | 0.84 |
| Magnesium (mmol/l) | continuous | 952 | 0.409 | 0.352 | 0.25 | 0.59 |
| QUICK/Thromboplastin time (%) | continuous | 924 | -0.004 | 0.002 | 0.047 | 0.33 |
| Thromboplastin time international normalized ratio | continuous | 946 | 0.142 | 0.137 | 0.30 | 0.62 |
| Partial thromboplastin time (s) | continuous | 948 | 0.031 | 0.011 | 0.005 | 0.08 |
| Fibrinogen acc. to Clauss (g/l) | continuous | 947 | -0.068 | 0.047 | 0.15 | 0.54 |
| Haemoglobin A1c (%) | continuous | 952 | 0.007 | 0.045 | 0.87 | 0.90 |
| Creatinine kinase, total (µkatal/l) | continuous | 952 | 0.007 | 0.023 | 0.76 | 0.86 |
| Lactate dehydrogenase (µkatal/l) | continuous | 952 | -0.103 | 0.075 | 0.17 | 0.55 |
| Leukocytes (Gpt/l) | continuous | 951 | -0.017 | 0.019 | 0.37 | 0.64 |
| Erythrocytes (tpt/l) | continuous | 951 | 0.033 | 0.091 | 0.71 | 0.86 |
| Haemoglobin (mmol/l) | continuous | 951 | -0.083 | 0.051 | 0.11 | 0.53 |
| Haematocrit | continuous | 951 | -1.266 | 1.138 | 0.27 | 0.59 |
| Mean corpuscular volume (fl) | continuous | 951 | -0.020 | 0.008 | 0.01 | 0.14 |
| Mean corpuscular haemoglobin (fmol) | continuous | 951 | -1.038 | 0.340 | 0.002 | 0.053 |
| Mean corpuscular haemoglobin concentration (mmol/l) | continuous | 951 | -0.089 | 0.057 | 0.12 | 0.54 |
| Red cell distribution width (%) | continuous | 951 | 0.038 | 0.039 | 0.33 | 0.64 |
| Thrombocytes (gpt/l) | continuous | 951 | 0.001 | 0.001 | 0.25 | 0.59 |
| Mean platelet volume (fl) | continuous | 951 | 0.009 | 0.027 | 0.75 | 0.86 |
| **Triglycerides (total) (mmol/l)** | **continuous** | **952** | **-0.091** | **0.026** | **<0.001** | **0.02** |
| Cholesterol (total) (mmol/l) | continuous | 950 | -0.056 | 0.030 | 0.067 | 0.41 |
| LDL-cholesterol (mmol/l) | continuous | 951 | -0.038 | 0.035 | 0.28 | 0.60 |
| HDL-cholesterol (mmol/l) | continuous | 951 | 0.104 | 0.094 | 0.26 | 0.59 |
| Alanine aminotransferase (µkatal/l) | continuous | 952 | -0.177 | 0.126 | 0.16 | 0.54 |
| Aspartate aminotransferase (µkatal/l) | continuous | 950 | -0.147 | 0.194 | 0.45 | 0.64 |
| Serum amylase (alpha-amylase) (μkatal/l) | continuous | 952 | -0.119 | 0.081 | 0.14 | 0.54 |
| Gamma-glutamyl transferase (µkatal/l) | continuous | 952 | -0.045 | 0.050 | 0.38 | 0.64 |
| Lipase (μkatal/l) | continuous | 952 | -0.010 | 0.028 | 0.72 | 0.86 |
| Thyroid-stimulating hormone (mU/l) | continuous | 951 | -0.097 | 0.040 | 0.017 | 0.17 |
| Folic acid (ng/ml) | continuous | 949 | 0.004 | 0.005 | 0.41 | 0.64 |
| Anti-thyroid peroxidase antibody (U/ml) | continuous | 945 | -0.0002 | 0.0002 | 0.25 | 0.59 |
| ***Somatometric variables*** |  |  |  |  |  |  |
| Height (cm) | continuous | 951 | -0.011 | 0.005 | 0.03 | 0.26 |
| Weight (kg) | continuous | 951 | -0.003 | 0.002 | 0.16 | 0.54 |
| Waist circumference (cm) | continuous | 951 | -0.005 | 0.003 | 0.08 | 0.42 |
| Hip circumference (cm) | continuous | 951 | -0.003 | 0.003 | 0.41 | 0.64 |

B, beta-coefficients from linear regression models; N, sample size; SE, standard error. * adjusted for multiple testing according to the Benjamini-Hochberg method (for multicategorical exposures, p values from the global test were considered)

**Supplementary Table 9. Linear regression model results evaluating associations of various variables with saliva levels of phenylacetate.**

| Variable | Level | N | B | SE | P value | q value* |
| --- | --- | --- | --- | --- | --- | --- |
| ***Medical diseases*** |  |  |  |  |  |  |
| Smoking (ref. never smoker) | Ex-smoker | 971 | -0.134 | 0.072 | 0.07 |  |
|  | Current smoker |  | -0.191 | 0.090 | 0.03 | 0.23 |
| Known or diagnosed diabetes mellitus | no/yes | 934 | 0.165 | 0.117 | 0.16 | 0.41 |
| Known diabetes mellitus | no/yes | 931 | 0.267 | 0.130 | 0.04 | 0.23 |
| Self-reported general state of health (Excellent or very good, ref.) | good | 972 | -0.181 | 0.077 | 0.02 |  |
|  | less good or bad |  | -0.117 | 0.121 | 0.33 | 0.23 |
| Has a raised or high blood pressure (hypertension) been determined medically since the last SHIP-study? | no/yes | 970 | 0.133 | 0.069 | 0.06 | 0.23 |
| Heart rate (bpm) | continuous | 969 | 0.002 | 0.003 | 0.58 | 0.79 |
| Diastolic blood pressure (mmHg) | continuous | 967 | 0.005 | 0.003 | 0.12 | 0.34 |
| Systolic blood pressure (mmHg) | continuous | 965 | 0.005 | 0.002 | 0.02 | 0.19 |
| Do you often suffer from a bad taste in your mouth? | no/yes | 971 | 0.161 | 0.091 | 0.08 | 0.24 |
| Do you often suffer from a bad breath? | no/yes | 912 | 0.179 | 0.095 | 0.06 | 0.23 |
| Did you suffer more than once from pancreatitis? | no/yes | 965 | -0.690 | 0.375 | 0.07 | 0.23 |
| Have you had one of the following medical problems (diseases) within the last 12 months? |  |  |  |  |  |  |
| Varicose veins, thrombosis, phlebitis | no/yes | 970 | 0.005 | 0.121 | 0.97 | 0.97 |
| Inflammatory skin diseases (e.g. acne) | no/yes | 967 | -0.341 | 0.112 | 0.002 | 0.06 |
| degeneration of the joints, e.g. arthrosis of the hip, knee, shoulder, or foot joints | no/yes | 966 | -0.207 | 0.078 | 0.008 | 0.11 |
| inflammatory joint diseases, e.g. chronic polyarthritis | no/yes | 961 | -0.172 | 0.174 | 0.32 | 0.59 |
| osteoporosis, i.e. reduced bone density | no/yes | 962 | 0.021 | 0.189 | 0.91 | 0.95 |
| gastritis | no/yes | 967 | -0.253 | 0.175 | 0.15 | 0.41 |
| hepatitis | no/yes | 971 | 1.224 | 0.571 | 0.03 | 0.23 |
| fatty liver | no/yes | 968 | -0.009 | 0.121 | 0.94 | 0.95 |
| increased level of blood lipids (cholesterol, triglycerides) | no/yes | 963 | 0.070 | 0.081 | 0.39 | 0.60 |
| gout or increased uric acid | no/yes | 964 | 0.103 | 0.130 | 0.43 | 0.64 |
| chronic bronchitis, i.e. coughing phlegm in the morning on most days, at least 3 months within the last 2 years? | no/yes | 971 | -0.045 | 0.168 | 0.79 | 0.91 |
| Have you ever had cancer? | no/yes | 969 | -0.165 | 0.147 | 0.26 | 0.51 |
| Do you suffer from a medically determined chronic lung disease? | no/yes | 971 | 0.204 | 0.170 | 0.23 | 0.46 |
| Do you suffer from psoriasis? | no/yes | 969 | 0.200 | 0.156 | 0.21 | 0.43 |
| Have you suffered from gastritis within the last six months? | no/yes | 966 | -0.068 | 0.184 | 0.71 | 0.89 |
| Have you ever had a stomach (gastric) ulcer? | no/yes | 969 | 0.095 | 0.119 | 0.43 | 0.64 |
| ***Laboratory variables*** |  |  |  |  |  |  |
| Sodium (mmol/l) | continuous | 972 | 0.018 | 0.014 | 0.20 | 0.43 |
| Potassium (mmol/l) | continuous | 972 | -0.116 | 0.087 | 0.18 | 0.42 |
| Serum creatinine (µmol/l) | continuous | 972 | -0.004 | 0.002 | 0.06 | 0.23 |
| Serum uric acid (µmol/l) | continuous | 972 | -0.0005 | 0.001 | 0.37 | 0.60 |
| Serum glucose (mmol/l) | continuous | 972 | 0.011 | 0.023 | 0.64 | 0.84 |
| Cystatin C (mg/l) | continuous | 971 | -0.633 | 0.269 | 0.02 | 0.19 |
| Serum calcium, total (mmol/l) | continuous | 972 | -0.080 | 0.335 | 0.81 | 0.92 |
| Magnesium (mmol/l) | continuous | 972 | -0.358 | 0.349 | 0.31 | 0.59 |
| QUICK/Thromboplastin time (%) | continuous | 944 | -0.003 | 0.002 | 0.17 | 0.41 |
| Thromboplastin time international normalized ratio | continuous | 966 | 0.179 | 0.137 | 0.19 | 0.43 |
| Partial thromboplastin time (s) | continuous | 968 | 0.009 | 0.011 | 0.39 | 0.60 |
| Fibrinogen acc. to Clauss (g/l) | continuous | 967 | 0.033 | 0.047 | 0.48 | 0.69 |
| Haemoglobin A1c (%) | continuous | 972 | -0.027 | 0.044 | 0.54 | 0.75 |
| Creatinine kinase, total (µkatal/l) | continuous | 972 | 0.015 | 0.023 | 0.51 | 0.72 |
| Lactate dehydrogenase (µkatal/l) | continuous | 972 | -0.013 | 0.074 | 0.86 | 0.94 |
| Leukocytes (Gpt/l) | continuous | 971 | 0.036 | 0.018 | 0.048 | 0.23 |
| Erythrocytes (tpt/l) | continuous | 971 | 0.141 | 0.090 | 0.12 | 0.34 |
| Haemoglobin (mmol/l) | continuous | 971 | 0.064 | 0.051 | 0.21 | 0.43 |
| Haematocrit | continuous | 971 | 2.422 | 1.129 | 0.03 | 0.23 |
| Mean corpuscular volume (fl) | continuous | 971 | 0.002 | 0.008 | 0.79 | 0.91 |
| Mean corpuscular haemoglobin (fmol) | continuous | 971 | -0.298 | 0.338 | 0.38 | 0.60 |
| Mean corpuscular haemoglobin concentration (mmol/l) | continuous | 971 | -0.118 | 0.056 | 0.04 | 0.23 |
| Red cell distribution width (%) | continuous | 971 | 0.014 | 0.039 | 0.71 | 0.89 |
| Thrombocytes (gpt/l) | continuous | 971 | 0.001 | 0.001 | 0.38 | 0.60 |
| Mean platelet volume (fl) | continuous | 971 | 0.002 | 0.027 | 0.94 | 0.95 |
| Triglycerides (total) (mmol/l) | continuous | 972 | 0.023 | 0.026 | 0.39 | 0.60 |
| Cholesterol (total) (mmol/l) | continuous | 970 | 0.055 | 0.030 | 0.07 | 0.23 |
| LDL-cholesterol (mmol/l) | continuous | 971 | 0.063 | 0.035 | 0.07 | 0.23 |
| HDL-cholesterol (mmol/l) | continuous | 971 | -0.049 | 0.092 | 0.60 | 0.80 |
| Alanine aminotransferase (µkatal/l) | continuous | 972 | 0.041 | 0.122 | 0.74 | 0.90 |
| Aspartate aminotransferase (µkatal/l) | continuous | 970 | -0.051 | 0.172 | 0.77 | 0.91 |
| Serum amylase (alpha-amylase) (μkatal/l) | continuous | 972 | -0.146 | 0.080 | 0.07 | 0.23 |
| Gamma-glutamyl transferase (µkatal/l) | continuous | 972 | 0.006 | 0.030 | 0.84 | 0.94 |
| Lipase (μkatal/l) | continuous | 972 | -0.038 | 0.027 | 0.17 | 0.41 |
| Thyroid-stimulating hormone (mU/l) | continuous | 971 | 0.015 | 0.040 | 0.72 | 0.89 |
| Folic acid (ng/ml) | continuous | 969 | 0.001 | 0.005 | 0.87 | 0.94 |
| Anti-thyroid peroxidase antibody (U/ml) | continuous | 965 | 0.00003 | 0.0002 | 0.88 | 0.94 |
| ***Somatometric variables*** |  |  |  |  |  |  |
| Height (cm) | continuous | 971 | -0.005 | 0.005 | 0.34 | 0.60 |
| Weight (kg) | continuous | 971 | 0.007 | 0.002 | 0.004 | 0.06 |
| **Waist circumference (cm)** | **continuous** | **971** | **0.010** | **0.003** | **<0.001** | **0.032** |
| Hip circumference (cm) | continuous | 971 | 0.010 | 0.003 | 0.003 | 0.06 |

B, beta-coefficients from linear regression models; N, sample size; SE, standard error. * adjusted for multiple testing according to the Benjamini-Hochberg method (for multicategorical exposures, p values from the global test were considered)

**Supplementary Table 10. Linear regression model results evaluating associations of various variables with saliva levels of Dimethylarginine (SDMA + ADMA).**

| Variable | Level | N | B | SE | P value | q value* |
| --- | --- | --- | --- | --- | --- | --- |
| ***Medical diseases*** |  |  |  |  |  |  |
| Smoking (ref. never smoker) | Ex-smoker | 944 | 0.043 | 0.073 | 0.55 |  |
|  | Current smoker |  | 0.124 | 0.091 | 0.17 | 0.66 |
| Known or diagnosed diabetes mellitus | no/yes | 911 | 0.200 | 0.116 | 0.09 | 0.24 |
| Known diabetes mellitus | no/yes | 908 | 0.229 | 0.130 | 0.08 | 0.23 |
| Self-reported general state of health (Excellent or very good, ref.) | good | 945 | 0.012 | 0.077 | 0.88 |  |
|  | less good or bad |  | 0.186 | 0.122 | 0.13 | 0.54 |
| Has a raised or high blood pressure (hypertension) been determined medically since the last SHIP-study? | no/yes | 943 | 0.151 | 0.069 | 0.03 | 0.14 |
| Heart rate (bpm) | continuous | 942 | -0.001 | 0.003 | 0.86 | 0.93 |
| Diastolic blood pressure (mmHg) | continuous | 940 | 0.002 | 0.003 | 0.55 | 0.78 |
| Systolic blood pressure (mmHg) | continuous | 938 | 0.004 | 0.002 | 0.048 | 0.19 |
| Do you often suffer from a bad taste in your mouth? | no/yes | 944 | 0.016 | 0.091 | 0.86 | 0.93 |
| Do you often suffer from a bad breath? | no/yes | 885 | -0.043 | 0.094 | 0.64 | 0.82 |
| Did you suffer more than once from pancreatitis? | no/yes | 938 | -0.151 | 0.372 | 0.69 | 0.82 |
| Have you had one of the following medical problems (diseases) within the last 12 months? |  |  |  |  |  |  |
| Varicose veins, thrombosis, phlebitis | no/yes | 943 | 0.231 | 0.121 | 0.06 | 0.21 |
| Inflammatory skin diseases (e.g. acne) | no/yes | 940 | -0.150 | 0.113 | 0.18 | 0.41 |
| degeneration of the joints, e.g. arthrosis of the hip, knee, shoulder, or foot joints | no/yes | 939 | -0.055 | 0.080 | 0.49 | 0.74 |
| inflammatory joint diseases, e.g. chronic polyarthritis | no/yes | 934 | -0.062 | 0.172 | 0.72 | 0.84 |
| osteoporosis, i.e. reduced bone density | no/yes | 935 | 0.181 | 0.192 | 0.35 | 0.64 |
| gastritis | no/yes | 940 | 0.071 | 0.174 | 0.68 | 0.82 |
| hepatitis | no/yes | 944 | -0.007 | 0.568 | 0.99 | 0.99 |
| fatty liver | no/yes | 941 | 0.302 | 0.120 | 0.01 | 0.07 |
| increased level of blood lipids (cholesterol, triglycerides) | no/yes | 936 | 0.056 | 0.082 | 0.49 | 0.74 |
| gout or increased uric acid | no/yes | 938 | 0.326 | 0.130 | 0.01 | 0.07 |
| chronic bronchitis, i.e. coughing phlegm in the morning on most days, at least 3 months within the last 2 years? | no/yes | 943 | 0.332 | 0.169 | 0.05 | 0.19 |
| Have you ever had cancer? | no/yes | 942 | -0.066 | 0.145 | 0.65 | 0.82 |
| Do you suffer from a medically determined chronic lung disease? | no/yes | 944 | 0.108 | 0.174 | 0.54 | 0.78 |
| Do you suffer from psoriasis? | no/yes | 942 | 0.076 | 0.155 | 0.62 | 0.81 |
| Have you suffered from gastritis within the last six months? | no/yes | 939 | 0.150 | 0.185 | 0.42 | 0.68 |
| Have you ever had a stomach (gastric) ulcer? | no/yes | 942 | 0.093 | 0.118 | 0.43 | 0.68 |
| ***Laboratory variables*** |  |  |  |  |  |  |
| Sodium (mmol/l) | continuous | 945 | 0.036 | 0.014 | 0.01 | 0.07 |
| Potassium (mmol/l) | continuous | 945 | 0.091 | 0.087 | 0.29 | 0.58 |
| Serum creatinine (µmol/l) | continuous | 945 | 0.001 | 0.002 | 0.58 | 0.81 |
| Serum uric acid (µmol/l) | continuous | 945 | 0.0003 | 0.0005 | 0.60 | 0.82 |
| Serum glucose (mmol/l) | continuous | 945 | 0.013 | 0.023 | 0.55 | 0.78 |
| Cystatin C (mg/l) | continuous | 944 | 0.686 | 0.268 | 0.01 | 0.07 |
| Serum calcium, total (mmol/l) | continuous | 945 | -0.024 | 0.336 | 0.94 | 0.98 |
| Magnesium (mmol/l) | continuous | 945 | -0.931 | 0.348 | 0.007 | 0.07 |
| QUICK/Thromboplastin time (%) | continuous | 917 | -0.004 | 0.002 | 0.096 | 0.24 |
| Thromboplastin time international normalized ratio | continuous | 939 | 0.247 | 0.137 | 0.071 | 0.23 |
| Partial thromboplastin time (s) | continuous | 941 | 0.017 | 0.011 | 0.12 | 0.29 |
| **Fibrinogen acc. to Clauss (g/l)** | **continuous** | **940** | **0.151** | **0.046** | **0.001** | **0.019** |
| Haemoglobin A1c (%) | continuous | 945 | 0.037 | 0.044 | 0.40 | 0.66 |
| Creatinine kinase, total (µkatal/l) | continuous | 945 | 0.007 | 0.023 | 0.75 | 0.85 |
| Lactate dehydrogenase (µkatal/l) | continuous | 945 | 0.156 | 0.074 | 0.036 | 0.16 |
| **Leukocytes (Gpt/l)** | **continuous** | **944** | **0.077** | **0.018** | **<0.001** | **0.002** |
| Erythrocytes (tpt/l) | continuous | 944 | 0.155 | 0.090 | 0.087 | 0.24 |
| Haemoglobin (mmol/l) | continuous | 944 | 0.049 | 0.051 | 0.34 | 0.64 |
| Haematocrit | continuous | 944 | 2.005 | 1.130 | 0.076 | 0.23 |
| Mean corpuscular volume (fl) | continuous | 944 | -0.002 | 0.008 | 0.76 | 0.85 |
| Mean corpuscular haemoglobin (fmol) | continuous | 944 | -0.434 | 0.338 | 0.20 | 0.44 |
| Mean corpuscular haemoglobin concentration (mmol/l) | continuous | 944 | 0.101 | 0.056 | 0.074 | 0.23 |
| **Red cell distribution width (%)** | **continuous** | **944** | **0.131** | **0.039** | **<0.001** | **0.019** |
| Thrombocytes (gpt/l) | continuous | 944 | 0.001 | 0.001 | 0.26 | 0.54 |
| Mean platelet volume (fl) | continuous | 944 | 0.013 | 0.027 | 0.64 | 0.82 |
| Triglycerides (total) (mmol/l) | continuous | 945 | -0.022 | 0.026 | 0.39 | 0.66 |
| Cholesterol (total) (mmol/l) | continuous | 943 | -0.009 | 0.030 | 0.76 | 0.85 |
| LDL-cholesterol (mmol/l) | continuous | 944 | 0.015 | 0.035 | 0.67 | 0.82 |
| HDL-cholesterol (mmol/l) | continuous | 944 | -0.197 | 0.092 | 0.032 | 0.15 |
| Alanine aminotransferase (µkatal/l) | continuous | 945 | 0.171 | 0.121 | 0.16 | 0.38 |
| Aspartate aminotransferase (µkatal/l) | continuous | 943 | 0.289 | 0.171 | 0.091 | 0.24 |
| Serum amylase (alpha-amylase) (μkatal/l) | continuous | 945 | -0.185 | 0.080 | 0.021 | 0.11 |
| Gamma-glutamyl transferase (µkatal/l) | continuous | 945 | 0.026 | 0.030 | 0.38 | 0.66 |
| Lipase (μkatal/l) | continuous | 945 | -0.028 | 0.027 | 0.30 | 0.58 |
| Thyroid-stimulating hormone (mU/l) | continuous | 944 | -0.003 | 0.044 | 0.95 | 0.98 |
| Folic acid (ng/ml) | continuous | 942 | 0.004 | 0.005 | 0.94 | 0.98 |
| Anti-thyroid peroxidase antibody (U/ml) | continuous | 940 | 0.000 | 0.0002 | 0.98 | 0.99 |
| ***Somatometric variables*** |  |  |  |  |  |  |
| Height (cm) | continuous | 944 | 0.013 | 0.005 | 0.015 | 0.09 |
| **Weight (kg)** | **continuous** | **944** | **0.008** | **0.002** | **<0.001** | **0.019** |
| **Waist circumference (cm)** | **continuous** | **944** | **0.008** | **0.003** | **0.003** | **0.047** |
| Hip circumference (cm) | continuous | 944 | 0.009 | 0.003 | 0.00 | 0.07 |

B, beta-coefficients from linear regression models; N, sample size; SE, standard error. * adjusted for multiple testing according to the Benjamini-Hochberg method (for multicategorical exposures, p values from the global test were considered)

**Supplementary Table 11.** **Description of genotyping and imputation.**

| Study | Genotyping chip and version | Number of study participants | Calling algorithm | SNP exclusions (pre-imputation QC) | Sample exclusion (pre-imputation QC) | Number of samples  (post QC) | Prephasing/  Imputation | SNP count (post imputation) |
| --- | --- | --- | --- | --- | --- | --- | --- | --- |
| SHIP | Affymetrix Genome-Wide Human SNP Array 6.0 | 4,308 | Birdseed2 | pHWE < 10^-4^, call rate < 95% or monomorphic SNVs | call rate < 92%, PCA outliers, duplicate samples (by IBS) or reported/genotyped gender mismatch | 4,070 | Imputation performed using Eagle v2.3 + and Minimac3 against the HRC (v r1.1 2016) reference panel on the Michigan Imputation server. | 40,356,094 |

**Supplementary Table 12.** **Overview on metabolites linked to 5-year tooth loss.**

| Metabolite | Class | Origin | Potential biological functions |
| --- | --- | --- | --- |
| 2-pyrrolidineacetic acid | Amino acid, nonproteinogenic | Bacteria/Host (bacterial product; exogenous compound) | Occurred in cured tobacco leaves (32), whose main active substance nicotine is known to have noxious effects on periodontal tissues. Higher frequency in current smokers, positively associated with WBC and correlated to 3-phenylpropionate, suggesting bacterial involvement. |
| Butyrylputrescine | Polyamine with a butyryl residual | Bacteria (degradation product of the amino acid arginine via ornithine | Different modifications of polyamines affect their availability in cells. However, putrescines equipped with a butyryl residual have not been described in the literature so far. Polyamines stimulate bacterial growth and are stress protective (39). Putrescine was increased in GCF samples of periodontitis patients and high PISA scores (18,35). |
| N-acetyl-cadaverine | Polyamine, acetylated | Bacteria (cadaverine is a degradation product of the amino acid lysine) | Polyamines have various functions, including stress protection, cell growth stimulation and cell proliferation (39,72). Cadaverine was significantly associated with periodontal inflammation (35,42). The acetylation of polyamines maintains their cellular concentration according to the metabolic need (39). In acetylated form they do not substitute for their parent molecules (73). |
| N,N-dimethyl-5-aminovalerate | Fatty acid (pentanoic acid with an amino substituent at C-5 and two methyl groups) | Bacteria (methylated degradation product of the amino acid lysine) | Methylated modification of 5-aminovalearte, a bacterial lysine degradation product. Bacteria metabolize lysine to cadaverine and finally 5-aminovalerate, which was related to increased PISA scores (35) and elevated in GCF of deep pockets (74). |
| dimethylarginine (SDMA + ADMA) | Amino acid derivate (Arginine), methylated | Bacteria (proteolysis of arginine-methylated proteins) | Growing bacterial colonization leads to an increased degradation of proteinaceous structures. Significantly interaction with N6,N6,N6-trimethyllysine. |
| N6,N6,N6-trimethyllysine | Amino acid derivate (Lysine), methylated | Bacteria (proteolysis of lysine methylated proteins/histones) | Increasing metabolic products of protein degradation are a sign of microbial overgrowth (16). Serves as a precursor of carnitine (75) which was found in periodontitis patients and is used by bacteria as energy source (17,46). |
| Phenylacetate | Carboxylic acid ester | Bacteria (degradation product of the aromatic amino acid phenylalanine) | Periodontal pathogenic bacteria use dipeptides and amino acids as nourishment for further growth. Aromatic amino acids were utilized by anaerobic bacteria, thereby increasing the number of degradation products (17). Phenylacetate was connected to periodontal pathogenic bacteria (76) and proposed as a potential biomarker for periodontitis, due to significant associations to increased PPD’s (16). |
| N-acetyltaurine | Amino sulfonic acid (Taurine), acetylated | Host (endogenous antioxidant mechanism, exogenous compound) | Product of taurine, which has antioxidant properties and supports the antioxidative status of chronic periodontitis patients (77,78). Antioxidants are required to protect the host tissue from ROS damage. ROS generation is a common mechanism against bacteria. |
| X – 23662 | Unknown | Unknown | Significantly interacts with cadaverine and N-acetyl-cadaverine (see Figure 4). |

WBC, white blood cell count; GCF, gingival crevicular fluid; PISA, periodontal inflamed surface areas; PPD, periodontal probing depth; ROS, reactive oxygen species.

**Supplementary Figures**

**
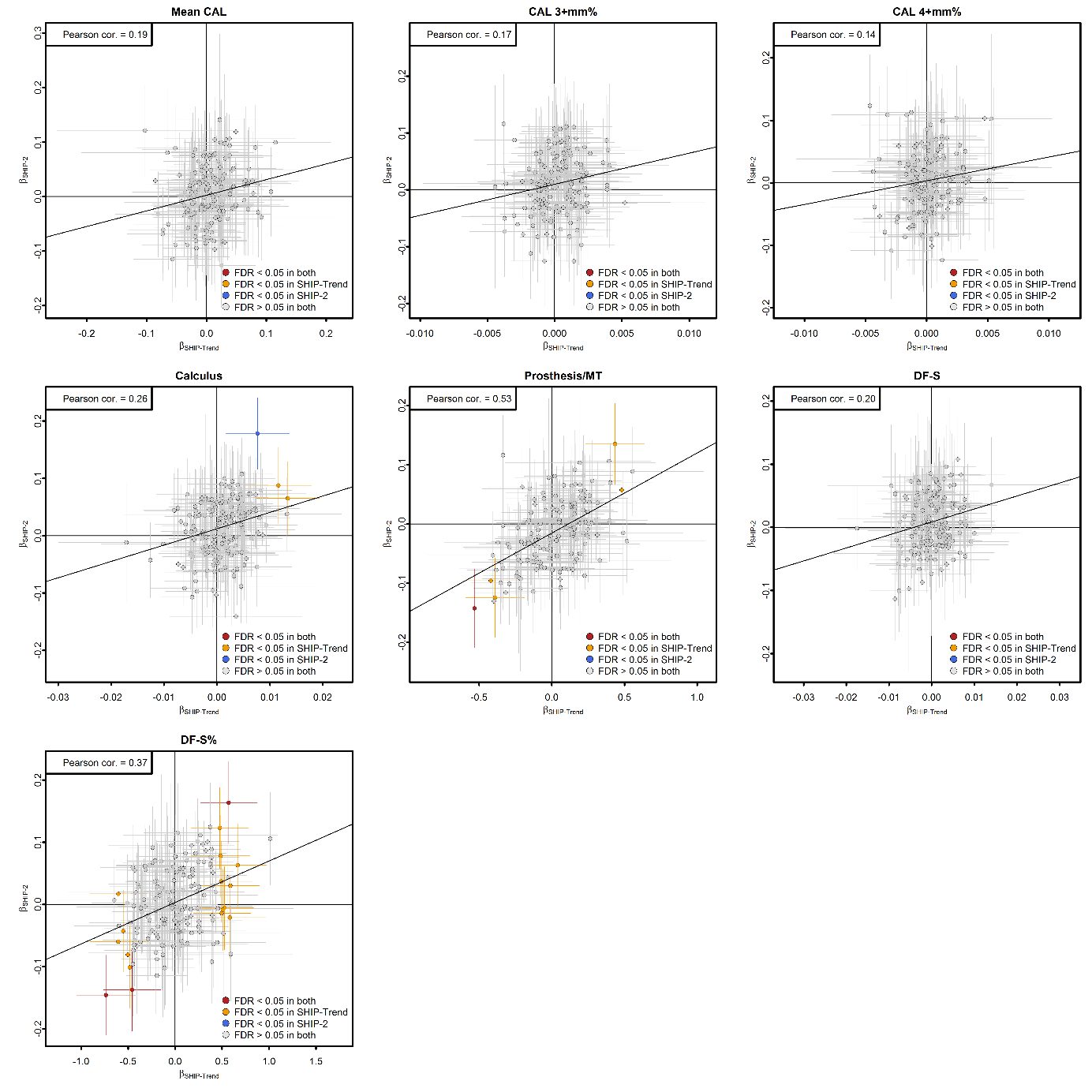
**

**Supplementary Figure 1. Point estimates comparison from regression models for selected dental variables between SHIP-2 and SHIP-Trend (16).** The remaining selected variables include Mean CAL, CAL 3+mm%, CAL 4+mm%, Calculus, Prosthesis/MT, DF-S and DF-S%. CAL, clinical attachment level; CAL 3+mm% and 4+mm%, percentages of sites with CAL ≥3 mm and ≥4 mm; DF-S and DF-S%, number and percentage of decayed or filled surfaces.

**
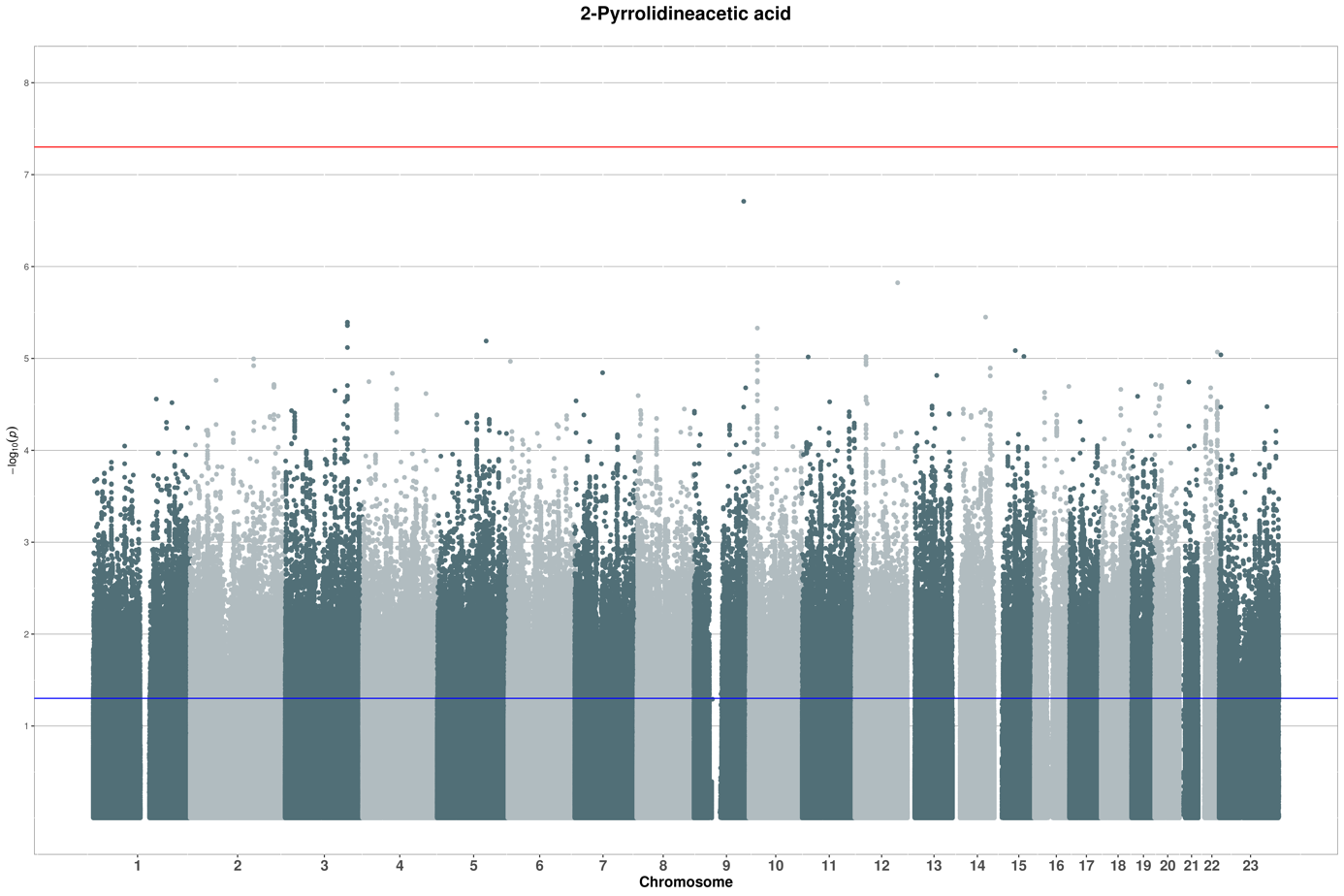
**

# Supplementary Figure 2. Manhattan-like plot displaying the results from a genome-wide analysis of 2-pyrr saliva levels. Manhattan-plot displaying the extent of association and genomic coordinates for single nucleotide polymorphisms (SNPs) signified by a dot. The horizontal red line represents the genome-wide significance threshold.

#
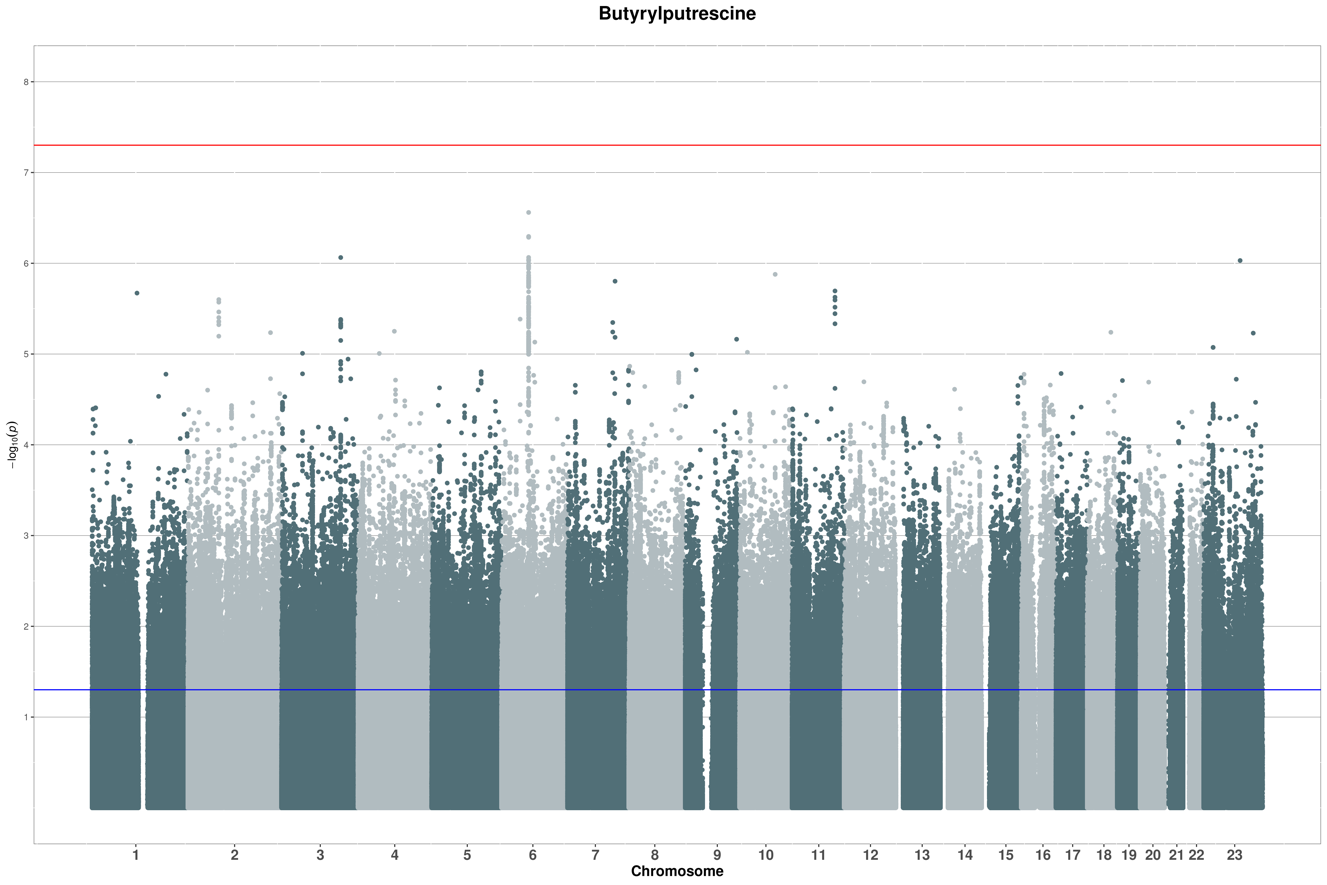


**Supplementary Figure 3. Manhattan-like plot displaying the results from a genome-wide analysis of butyrylputrescine saliva levels.** Manhattan-plot displaying the extent of associations and genomic coordinates for single nucleotide polymorphisms (SNPs) signified by a dot. The horizontal red line represents the genome-wide significance threshold.

**
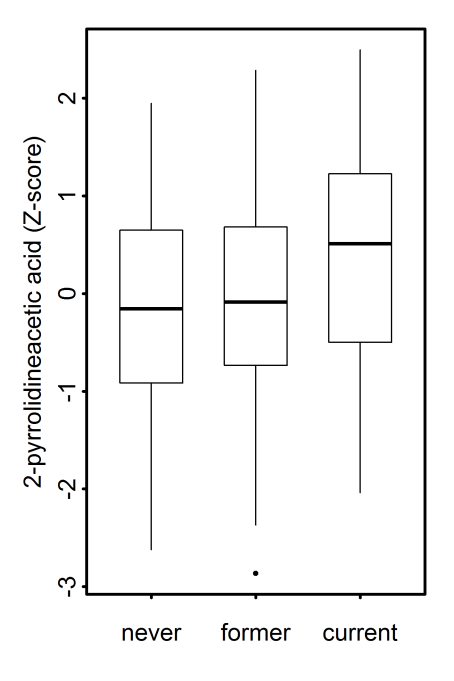
**

**Supplementary Figure 4. Distribution of 2-pyrrolidineacetic acid by smoking status.**
